# Liver Stiffness Rises Early in MASLD and Drives Inflammation, Lipid Dysmetabolism, and Fibrosis via Piezo1–YAP Mechanotransduction

**DOI:** 10.1002/advs.202519109

**Published:** 2026-01-04

**Authors:** Juan Ma, Ning Xie, Ziwei Wang, Xiru Liang, Yulong Han, Qiang Zhao, Ming Wang, Hongwei Lu, Wanyi Kou, William Alazawi, Jinhai Wang, Lu Li, Ning Liu, Na Liu, Haitao Shi, Feng Xu

**Affiliations:** ^1^ Department of Gastroenterology The Second Affiliated Hospital of Xi'an Jiaotong University Xi'an Shaanxi P. R. China; ^2^ The Key Laboratory of Biomedical Information Engineering of Ministry of Education School of Life Science and Technology Xi'an Jiaotong University Xi'an Shaanxi P. R. China; ^3^ Bioinspired Engineering and Biomechanics Center (BEBC) Xi'an Jiaotong University Xi'an Shaanxi P. R. China; ^4^ State Key Laboratory of Mechanics and Control for Aerospace Structures Nanjing University of Aeronautics and Astronautics Nanjing P. R. China; ^5^ Department of General Surgery The Second Affiliated Hospital of Xi'an Jiaotong University Xi'an Shaanxi P. R. China; ^6^ Barts Liver Centre Blizard Institute Queen Mary University of London London UK; ^7^ Department of Gastrointestinal Surgery Hainan General Hospital Hainan Affiliated Hospital of Hainan Medical University Haikou P. R. China; ^8^ Department of Gastroenterology Hainan General Hospital (Hainan Affiliated Hospital of Hainan Medical University) Haikou P. R. China

**Keywords:** lipid droplets, liver stiffness, mechanomedicine, metabolic dysfunction–associated steatotic liver disease, Piezo1, YAP

## Abstract

Metabolic dysfunction–associated steatotic liver disease (MASLD) is shaped by metabolic injury and tissue mechanics. This study investigated whether liver stiffening occurs early in MASLD and how extracellular matrix (ECM) mechanics interact with lipid droplet (LD) overload to promote inflammation, fibrogenesis, and lipid dysmetabolism. Clinical data, mouse models, and in vitro experiments are integrated. Liver stiffness shifted modestly with steatosis but increased substantially in the presence of inflammation. In a diet‐induced mouse model, liver stiffness increased before overt fibrosis. In cultured hepatocytes, stiff matrices combined with free fatty acid (FFA) induced steatosis synergistically amplified pro‐inflammatory and pro‐fibrotic signals, accompanied by cytoskeletal remodeling and nuclear deformation. YAP acted as a central mechanosensitive amplifier: stiffness drove YAP nuclear localization, and *YAP* knockdown blunted cytokine induction and fibrogenic gene expression under stiff + FFA conditions. Stiffness and LD overload jointly promoted lipogenesis and impaired lipophagy via YAP. Piezo1 is upregulated by stiff + FFA; its inhibition reduced Ca^2^⁺ influx and prevented YAP activation. Collectively, early liver stiffening, together with LD‐derived intracellular stress, engages a Piezo1–YAP axis that amplifies inflammation, fibrogenesis, and disordered lipid metabolism, consistent with a proposed feed‐forward loop mechanism accelerating MASLD progression.

## Graphical abstract

Progressive stiffening of the hepatic extracellular matrix and free fatty acid overload activate the mechanosensor Piezo1 in steatotic hepatocytes, inducing calcium influx and nuclear translocation of Yes‐associated protein. YAP‐dependent nuclear signaling further drives inflammatory and fibrotic gene programs, lipid metabolic reprogramming, and impaired lipophagy, forming a feed‐forward loop that amplifies matrix stiffening and disease progression.

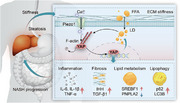

## 1 Introduction

Metabolic dysfunction‐associated steatotic liver disease (MASLD), recently reclassified from nonalcoholic fatty liver disease (NAFLD), has emerged as one of the most prevalent chronic liver disorders worldwide. It is estimated to affect more than one‐third of the adult population and is increasingly recognized as a major driver of liver‐related morbidity, mortality, and healthcare burden [1, 2, 3, 4]. MASLD comprises a broad histological spectrum, ranging from simple steatosis to metabolic dysfunction‐associated steatohepatitis (MASH, formerly NASH), with potential progression to advanced fibrosis, cirrhosis, and hepatocellular carcinoma (HCC) [5, 6, 7, 8]. The rapid global rise in obesity and metabolic syndrome is expected to further accelerate this trajectory, with cases of advanced disease projected to increase substantially by 2030 [9]. Despite heightened awareness and progress in therapeutic development, effective disease‐modifying interventions remain limited [10, 11].

Beyond canonical dyslipidemia, insulin resistance, and lipotoxicity, accumulating evidence highlights extracellular matrix (ECM) remodeling and tissue mechanics as active determinants of hepatocyte behavior [12, 13, 14, 15, 16]. Progressive ECM deposition and cross‐linking stiffen the parenchyma, a shift sensitively a biophysical alteration readily detected by elastography [17, 18]. Notably, mechanical abnormalities can emerge even before overt fibrosis and influence disease evolution [19]. For example, in pre‐cirrhotic NASH with type 2 diabetes, advanced glycation product (AGE)‐driven collagen remodeling increases ECM viscoelasticity without altering stiffness. This mechanical alteration promotes hepatocellular carcinoma through the integrin‐β1/tensin‐1/YAP signaling pathway. Inhibition of AGE formation mitigates these effects [15]. Similarly, early MASLD in patients with diabetes is already associated with AGE accumulation and increased viscoelasticity [20]. At the cellular level, lipid droplets (LDs), once regarded as inert metabolic depots, generate surface tension and rigidity sufficient to compress nuclei and disrupt cytoskeletal architecture [21], revealing an intracellular source of mechanical stress that has yet to be integrated with extracellular cues [22, 23, 24]. How these extracellular and intracellular forces converge to drive lipid dysregulation, inflammation, and fibrosis remains poorly understood.

We hypothesized that early increases in liver stiffness synergize with metabolic stress to promote MASLD progression through a “stiffness/LD‐stress–Piezo1–YAP” axis. To examine this, population‐scale elastography data (>24 000 individuals), murine models, and tunable hydrogel systems were integrated to interrogate mechanical cues across biological scales. Liver stiffness increased from the steatosis stage onward and rose before overt histological fibrosis became detectable. Stiff matrices synergize with FFA overload to induce cytoskeletal and nuclear remodeling, LD hyper‐accumulation, and Piezo1‐dependent YAP activation, thereby establishing a feed‐forward loop linking mechanics to inflammation and fibrogenic signaling. This framework supports the potential use of elastography‐based early risk stratification and highlights stiffness‐modulating biomaterials and Piezo1/YAP‐targeted therapies as promising mechanomedicine‐informed strategies for early MASLD intervention.

## 2 Results

### 2.1 Liver Stiffening Emerges Early in MASLD and Correlates With Steatosis, Inflammation, and Fibrosis

**FIGURE 1:**
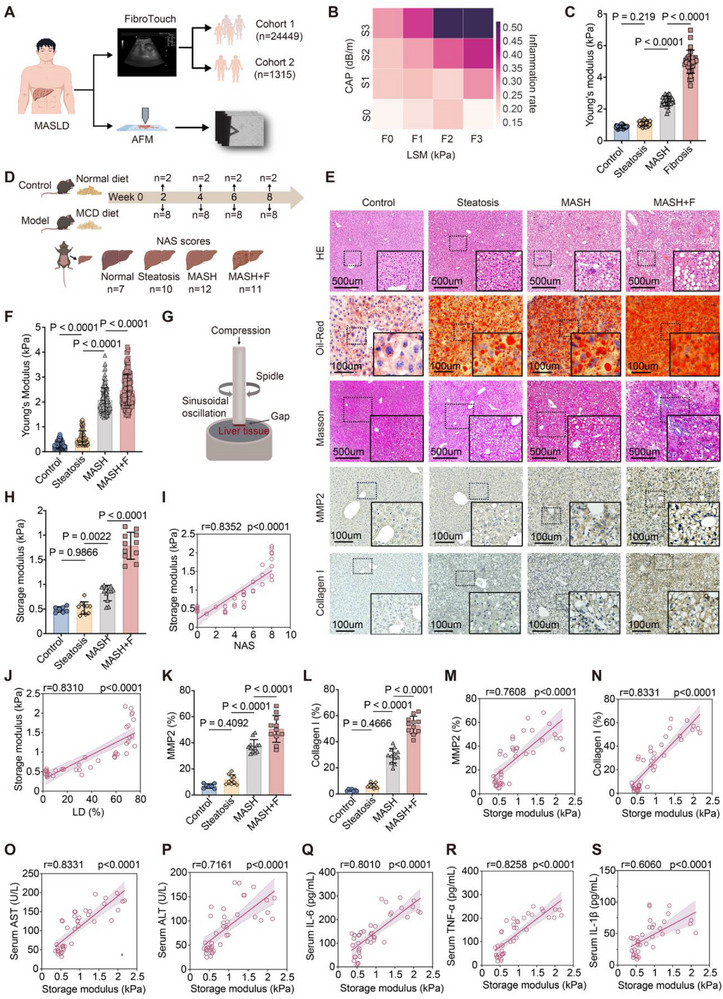
Hepatic tissue stiffening emerges early in MASLD progression and correlates with steatosis, inflammation, and fibrosis. (A) Schematic of study design. Two clinical cohorts (n = 24 449 and n = 1,315) underwent liver stiffness measurement (LSM) and controlled attenuation parameter (CAP) assessment, with surgical specimens used for atomic force microscopy (AFM) analysis. (B) Heatmap showing the relationship between CAP‐defined steatosis grade (S0–S3) and LSM‐defined fibrosis stage (F0–F3). (C) Young's modulus of liver tissue measured by AFM in control, NAFL, MASH, and fibrosis patients. (D) Experimental timeline of the methionine‐ and choline‐deficient (MCD) diet mouse model (n = 7–12 per group). (E) Representative histological staining of mouse liver tissues: hematoxylin–eosin (H&E), Oil Red O, Masson's trichrome, and immunohistochemistry (IHC) for MMP2 and collagen I. Scale bars as indicated. (F) Quantification of AFM‐derived Young's modulus across groups. (G) Schematic of the rheometry setup used for oscillatory shear measurements of liver tissue. (H) Storage modulus measured by rheometry in control and MCD‐fed mice. (I) Correlation of storage modulus with MASLD activity score (NAS). (J) Correlation of storage modulus with LD percentage. (K,L) Quantification of MMP2 and collagen I expression by IHC. (M,N) Correlation of storage modulus with MMP2 and collagen I expression. (O–S) Correlations of storage modulus with serum biochemical and inflammatory markers, including AST (O), ALT (P), IL‐6 (Q), TNF‐α (R), and IL‐1β (S). Data are shown as mean ± SD. Statistical significance was determined by one‐way ANOVA with post hoc tests for multiple comparisons or Spearman's correlation for associations.

To determine whether liver mechanical properties are altered at the earliest stages of MASLD, we analyzed two independent clinical cohorts (Figure 1A). In Cohort 1 (n = 24 449), LSM increased modestly with controlled attenuation parameter (CAP) defined steatosis severity (Figure S1A). In Cohort 2 (n = 1315), individuals with elevated aminotransferases (indicating probable inflammation) showed higher CAP and LSM than enzyme‐normal counterparts, and joint CAP–LSM distribution analysis revealed a rightward shift in those with inflammation (Figure 1B; Figure S1B,C). Tissue‐level measurements corroborated these clinical trends: AFM nanoindentation of surgical liver specimens (n = 5; ∼250 sites per specimen) demonstrated graded increases in Young's modulus from healthy livers to simple steatosis, steatohepatitis, and advanced fibrosis (Figure 1C). Together, these data indicated that stiffness elevations accompany early steatosis and extend through inflammatory and fibrotic stages, supporting the utility of noninvasive elastography to detect nascent mechanical alterations in MASLD.

To validate the clinical observations in a controlled setting, we established a diet‐induced mouse model that recapitulates distinct stages of disease progression. Mice fed the methionine‐ and choline‐deficient (MCD) diet were stratified by NAFLD activity score (NAS) to represent simple steatosis, steatohepatitis, and fibrosis (Figure 1D). Histology demonstrated increasing LD accumulation (Oil Red O staining), inflammatory cell infiltration (H&E), and septal fibrosis (Masson's trichrome) with advancing disease (Figure 1E). Consistent with these pathological changes, AFM and rheometry revealed progressive increases in the apparent Young's modulus and storage modulus G′ from healthy to steatotic to fibrotic livers (Figure 1F–H). Storage modulus correlated strongly with histological NAS (Figure 1I) and with the percentage of liver area occupied by LDs (Figure 1J). At the molecular level, matrix‐remodeling proteins (e.g., MMP2, collagen I) increased across disease stages by immunohistochemistry (Figure 1E,K,L) and positively correlated with tissue stiffness (storage modulus) (Figure 1M,N). Moreover, storage modulus was significantly associated with serum markers of hepatic injury (AST, ALT; Figure 1O,P) and circulating pro‐inflammatory cytokines (IL‐6, TNF‐α, IL‐1β; Figure 1Q–S). Collectively, these results indicated that hepatic stiffening arises early alongside steatosis and is amplified by inflammation and fibrosis, supporting concerted lipid–matrix interactions that may contribute to MASLD progression. Given the co‐elevation of CAP and LSM and their association with inflammatory markers in the clinical cohorts, we next investigated the mechanistic basis by which matrix stiffness interacts with lipid overload to regulate inflammatory and metabolic outputs.

### 2.2 ECM Stiffness and LD Accumulation Synergize to Elevate Pro‐Inflammatory and Pro‐Fibrotic Signaling in Hepatocytes

**FIGURE 2:**
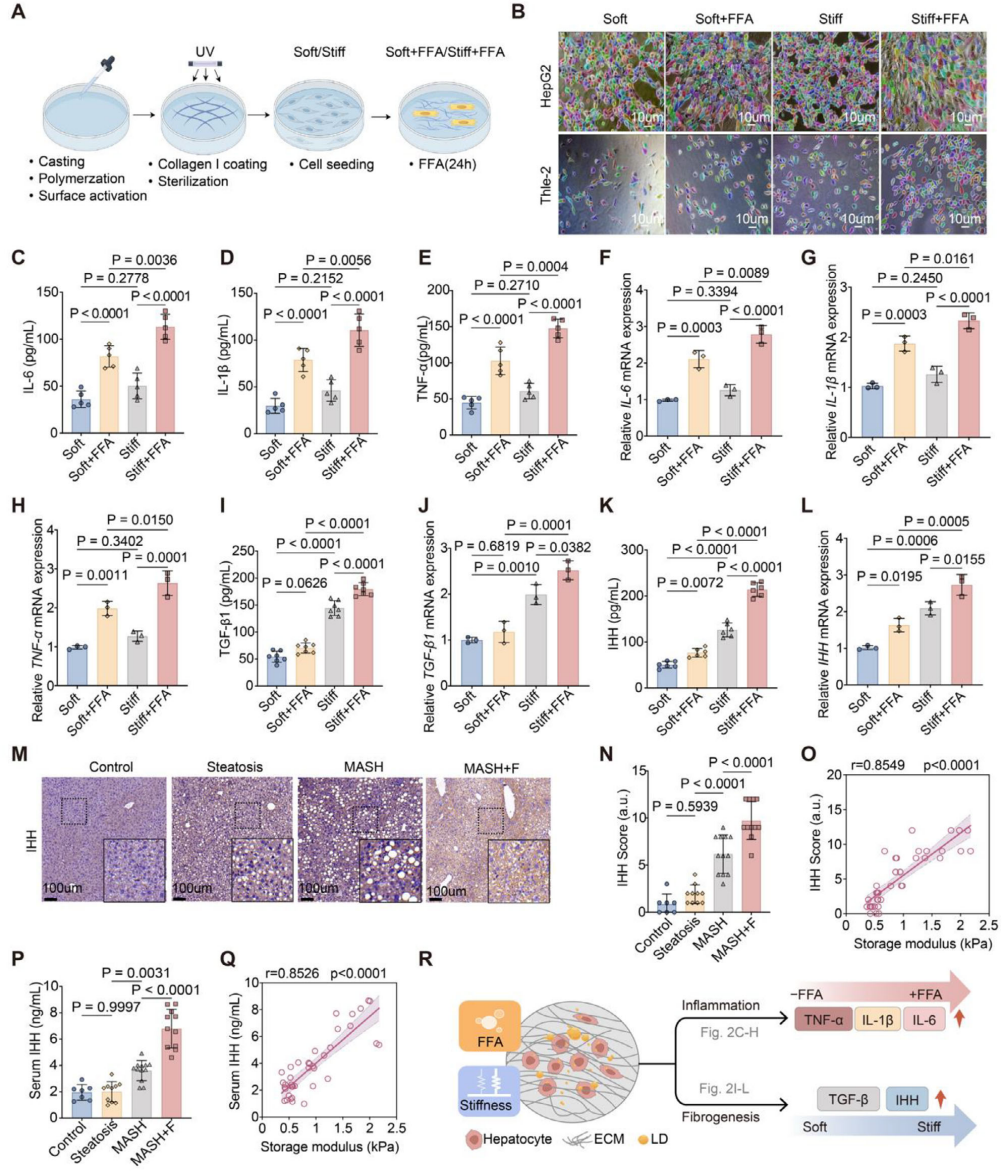
ECM stiffness and lipid overload synergize to enhance pro‐inflammatory and pro‐fibrotic signaling in hepatocytes. (A) Schematic of polyacrylamide hydrogel system simulating soft (∼0.5 kPa) and stiff (∼20 kPa) liver matrices with or without FFA treatment (oleate: palmitate, 2:1; 0.5 mm, 24 h). (B) Representative images of HepG2 and THLE‐2 cells showing stiffness‐ and FFA‐dependent morphological changes. Scale bars, 100 µm. (C–E) ELISA quantification of secreted IL‐6, IL‐1β, and TNF‐α in HepG2 cells (n = 3 per group). (F–H) Transcript levels of IL‐6, IL‐1β, and TNF‐α in HepG2 cells (n = 3 per group). (I,J) Protein and transcript levels of TGF‐β1 in HepG2 cells (n = 3 per group). (K,L) Protein and transcript levels of IHH in HepG2 cells (n = 3 per group). (M) Immunohistochemistry of IHH in mouse livers at different disease stages (Control, NAFL, MASH, MASH+F). Scale bars, 100 µm. (N–P) Quantification of hepatic IHH score and serum IHH levels across disease stages (n = 7–12 per group). (O–Q) Correlation of storage modulus with hepatic IHH score and serum IHH concentration (Spearman's r, *p <*0.0001). (R) Working model: LD accumulation dominantly induces inflammatory cytokines (IL‐6, IL‐1β, TNF‐α), while matrix stiffness drives fibrogenic mediators (TGF‐β1, IHH). Together, these cues synergize to accelerate the transition from inflammation to fibrosis. Data are mean ± SD (n = 3 independent experiments). Statistical significance was determined by one‐way ANOVA with post hoc tests for multiple comparisons or Spearman's correlation for associations.

Collagen I‐coated polyacrylamide hydrogels were used to model a normal “soft” liver matrix (∼0.5 kPa) versus a fibrotic “stiff” matrix (∼20 kPa), with or without FFA (OA: PA = 2:1 ratio, 0.5 mm total) to induce LD accumulation in THLE‐2 and HepG2 hepatocytes (Figure 2A). Bright‐field imaging with quantitative cell segmentation showed that on soft substrates, cells assumed a polygonal spread morphology with clear cell–cell boundaries and a dispersed arrangement, whereas higher stiffness induced marked cell elongation, alignment along a common axis, and tighter cell packing. Under soft conditions, FFA‐induced steatosis caused modest cell enlargement and irregular shape; in the context of high stiffness, the same FFA loading further enhanced cell elongation and alignment, exaggerating overall morphological changes (Figure 2B). Functionally, LD accumulation alone robustly induced IL‐6, IL‐1β, and TNF‐α, while stiffness alone was insufficient to trigger these cytokines. Strikingly, the combination of stiffness with LD loading markedly amplified inflammatory cytokine levels (*p <*0.0001), with LDs serving as the dominant driver of this response (Figure 2C–H). By contrast, pro‐fibrotic mediators TGF‐β1 and IHH were primarily stiffness‐driven and were further amplified by the presence of LDs. Consistently, hepatic and serum IHH levels increased with disease severity and correlated strongly with storage modulus in vivo (r >0.85, *p <*0.0001; Figure 2I–Q). Thus, LD overload dominated inflammatory induction, whereas stiffness predominantly activated fibrogenic programs; together, these cues acted synergistically to accelerate the transition from inflammation to fibrosis (Figure 2R).

### 2.3 ECM Stiffness Synergizes With LD Accumulation to Remodel Hepatocyte Cytoskeletal Architecture and Nuclear Morphology

**FIGURE 3:**
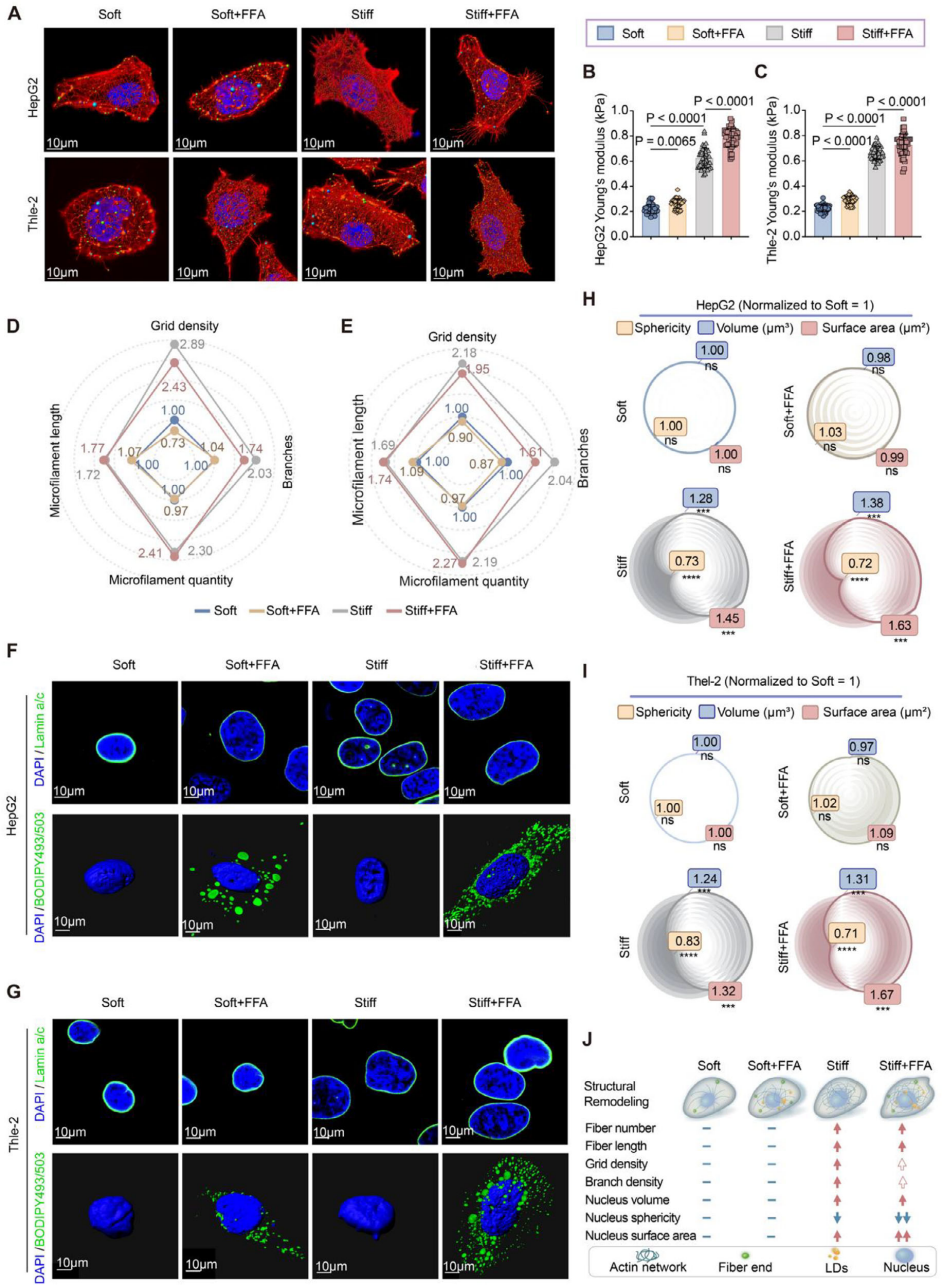
ECM stiffness synergizes with lipid overload to remodel hepatocyte cytoskeletal architecture and nuclear morphology. (A) Representative 3D reconstructions of HepG2 and THLE‐2 cells generated using Imaris software from confocal z‐stacks. Cells were cultured on soft (∼0.5 kPa) or stiff (∼20 kPa) collagen‐coated PA hydrogels, with or without FFA treatment (0.5 mm, oleate: palmitate 2:1). F‐actin filaments (phalloidin, red) and nuclei (DAPI, blue) were rendered in 3D. Filament tracing was performed to quantify actin fiber number, length, branching, and grid density, while nuclear geometry (volume, surface area, sphericity) was measured using the Imaris “Surface” module. Scale bars: 10 µm. (B,C) Quantification of apparent Young's modulus by AFM indentation in HepG2 (B) and THLE‐2 (C) cells under the indicated conditions. (D,E) Radar plots showing normalized microfilament architecture parameters, including filament length, number, branching, and grid density, in HepG2 (D) and THLE‐2 (E) cells. All parameters were normalized to the soft condition (= 1). (F,G) Immunofluorescence images of nuclear morphology (Lamin A/C, green) and LD accumulation (BODIPY 493/503, green) in HepG2 (F) and THLE‐2 (G) cells. Nuclei were counterstained with DAPI (blue). Scale bars: 10 µm. (H, I) Quantitative analysis of nuclear geometry in HepG2 (H) and THLE‐2 (I) cells, including sphericity, volume, and surface area, normalized to Soft = 1. Statistical significance was calculated relative to the soft group and is indicated by asterisks on the plots. Absence of asterisks denotes no significant difference. (J) Schematic summary of structural remodeling induced by ECM stiffness and FFA. Stiffness promotes cytoskeletal reorganization (increased fiber number/length, reduced branching), while LD accumulation exerts perinuclear compression. Combined stiff + FFA conditions result in pronounced actin alignment, nuclear deformation, and LD accumulation. Data are mean ± SD (n = 3 independent biological replicates). Statistical significance was determined by one‐way ANOVA followed by Tukey's post hoc test. ^*^ *p <*0.05, ^**^ *p <*0.01, ***p <0.001, ^****^ *p <*0.0001; ns, not significant.

Building on the morphological changes described above, we conducted high‐resolution confocal imaging of HepG2 and THLE‐2 cells (Figure S3A,B). Actin immunofluorescence z‐stacks were reconstructed in 3D using Imaris to enable quantitative morphometric analysis (Figure 3A). On stiff matrices, both cell types displayed prominent stress fibers and significantly increased apparent Young's modulus compared with soft substrates (Figures 3B,C). Quantitative analysis revealed that stiffness markedly increased F‐actin filament number and length, while reducing branching and grid density. LD loading alone induced only modest cytoskeletal changes. Strikingly, under stiff + FFA conditions, cytoskeletal remodeling was most pronounced, with extensive parallel stress‐fiber bundles and reduced network complexity, indicating a shift from dense intersecting filaments to highly ordered arrays (Figure 3D, E).

Given the intimate mechanical coupling between cytoskeleton and nucleus, we next assessed nuclear geometry by lamin A/C staining with 3D reconstruction (Figure 3F, G). Stiff substrates increased nuclear volume and surface area while reducing sphericity in both cell lines (Figure 3H, I). LD accumulation alone had minimal effects on nuclear morphology. However, under stiff + FFA conditions, HepG2 nuclei exhibited further enlargement and loss of sphericity, whereas THLE‐2 nuclei showed limited additional deformation. These cell‐type‐specific differences suggest distinct nuclear response thresholds between malignant (HepG2) and non‐malignant (THLE‐2) hepatocytes.

Together, these results demonstrate that ECM stiffness serves as the primary regulator of cytoskeletal reorganization and nuclear deformation, whereas LD accumulation acts as a perinuclear compressive force that reduced actin network connectivity. The combination of stiffness and steatosis thereby amplified cytoskeletal alignment and nuclear remodeling, driving distinct mechano‐metabolic adaptations in hepatocytes (Figure 3J).

### 2.4 YAP Mediates ECM Stiffness‐ and Lipid Overload‐Induced Pro‐Inflammatory and Pro‐Fibrotic Transcriptional Programs

**FIGURE 4:**
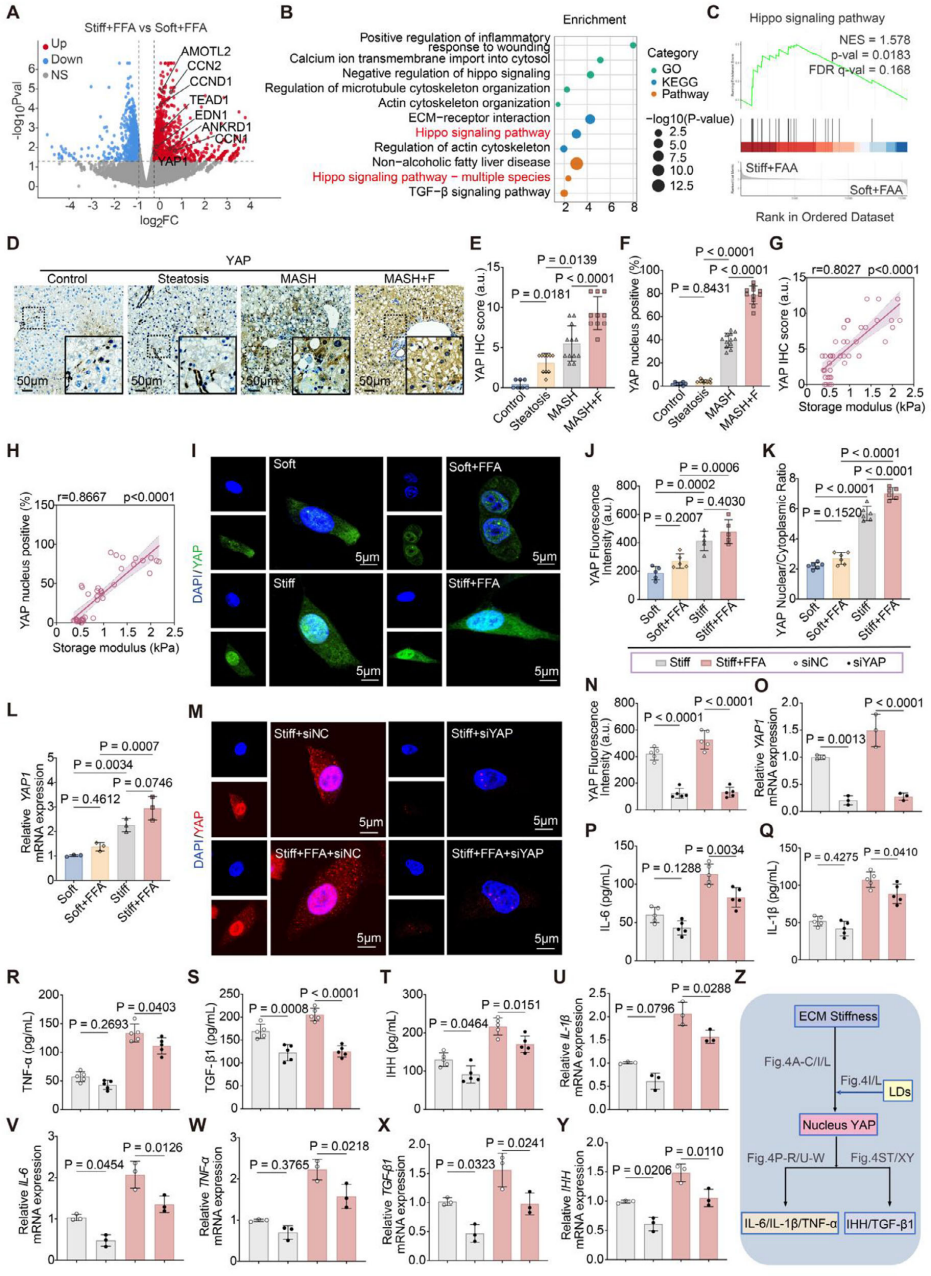
YAP mediates ECM stiffness‐ and lipid overload‐induced pro‐inflammatory and pro‐fibrotic transcriptional programs. (A) Volcano plot showing differential gene expression in HepG2 cells cultured on stiff hydrogels with FFA (stiff + FFA) versus soft hydrogels with FFA (soft + FFA). Classical YAP target genes (e.g., *AMOTL2, CCN2, CCND1, TEAD1, EDN1, ANKRD1*) were significantly upregulated in the stiff + FFA condition. Thresholds: |log_2_FC| > 1 and FDR <0.05. (B) GO/KEGG enrichment analysis based on the stiff + FFA versus soft + FFA comparison showing significant enrichment of pathways related to cytoskeletal organization, ECM–receptor interaction, and Hippo/YAP signaling. (C) GSEA further confirmed significant activation of the Hippo/YAP signaling pathway under stiff + FFA relative to soft + FFA conditions. (D) IHC staining of YAP in mouse liver tissues from control, Steatosis, MASH, and fibrotic MASH (MASH+F) groups. Scale bars: 50 µm. (E,F) Quantification of YAP IHC scores (E) and percentage of YAP nuclear‐positive hepatocytes (F) across mouse groups. (G,H) Correlations between YAP IHC scores (G) or percentage of YAP nuclear‐positive cells (H) and liver storage modulus in mouse liver tissues. (I–K) Immunofluorescence showing YAP localization in HepG2 cells. On soft hydrogels, YAP remains cytoplasmic (even with LD accumulation), whereas stiffness drives YAP nuclear translocation, further enhanced by FFA treatment. (J) Quantification of YAP fluorescence intensity; (K) Nucleus‐to‐cytoplasm (N/C) fluorescence ratio. Scale bars: 5 µm. (L) qPCR analysis of YAP1 mRNA levels under the indicated conditions. (M–N) siRNA knockdown of YAP reduced YAP nuclear accumulation and fluorescence intensity. Scale bars: 5 µm. (O–Y) Functional readouts of YAP activity. (P–R) Cytokine secretion (IL‐6, IL‐1β, TNF‐α) by ELISA under stiff + FFA versus controls, showing YAP‐dependent induction. (S,T) Fibrogenic mediators (TGF‐*β*1, IHH) measured by ELISA, predominantly stiffness‐driven but still YAP‐dependent. (U–Y) qPCR confirming corresponding changes in cytokine and fibrogenic gene expression. (Z) Schematic model: ECM stiffness promotes YAP nuclear translocation, activating pro‐fibrotic transcriptional programs; lipid overload recruits YAP as a transcriptional co‐activator, amplifying inflammatory signaling. Together, these synergistic inputs reinforce inflammation and fibrosis, accelerating MASLD progression. Data are mean ± SD (n = 3 independent biological replicates). Statistical significance was determined by one‐way ANOVA with Tukey's post hoc test or Spearman's correlation.

Transcriptomic profiling of HepG2 cells cultured under different conditions was performed. Pairwise comparison between stiff + FFA and soft + FFA (Figure 4A) showed marked upregulation of canonical YAP target genes (e.g., *AMOTL2, CCN2/CYR61, CCND1, ANKRD1, EDN1)*. GO and KEGG enrichment analyses revealed significant enrichment of pathways related to Hippo/YAP signaling and cytoskeletal organization (Figure 4B). Consistently, GSEA based on the stiff + FFA versus soft + FFA ranking further confirmed activation of the Hippo signaling pathway (Figure 4C). In mouse liver tissues, YAP expression and nuclear localization were low in healthy controls and simple steatosis, but robustly increased in MASH and further elevated in fibrotic MASH, correlating with rheometry‐measured storage modulus (Figure 4D–H). In vitro, YAP remained predominantly cytoplasmic on soft matrices (even with LD loading), whereas stiffness drove YAP nuclear translocation, which was further enhanced by FFA treatment (Figure 4I–K). qPCR analysis confirmed increased *YAP1* mRNA levels on stiff matrices, especially under stiff + FFA (Figure 4L), and siRNA knockdown of YAP reduced YAP nuclear accumulation and transcriptional activity (Figure 4M–O).

Functionally, YAP knockdown selectively blunted cytokine induction under stiff + FFA conditions (IL‐6, IL‐1β, TNF‐α; Figure 4P–R) but had no effect when stiffness or FFA were applied alone, indicating that maximal inflammatory output requires combined mechanical and metabolic inputs. By contrast, fibrogenic mediators (TGF‐β1, IHH) were predominantly stiffness‐driven and remained uniformly YAP‐dependent under all stiff conditions and were further amplified by LD overload (Figure 4S, T), with concordant alterations at the mRNA level (Figure 4U–Y). These data are consistent with a model (Figure 4Z) wherein ECM stiffness promotes YAP nuclear translocation to activate pro‐fibrotic transcriptional programs, while lipid overload engages YAP as a transcriptional co‐activator that potentiates inflammatory signaling. Together, these inputs form a synergistic circuit reinforcing inflammation and fibrosis, contributing to MASLD progression and positioning YAP as both an amplifier of inflammation and a central driver of fibrogenesis.

### 2.5 YAP Links Matrix Stiffness to Lipogenic Reprogramming and Lipophagy Suppression

**FIGURE 5:**
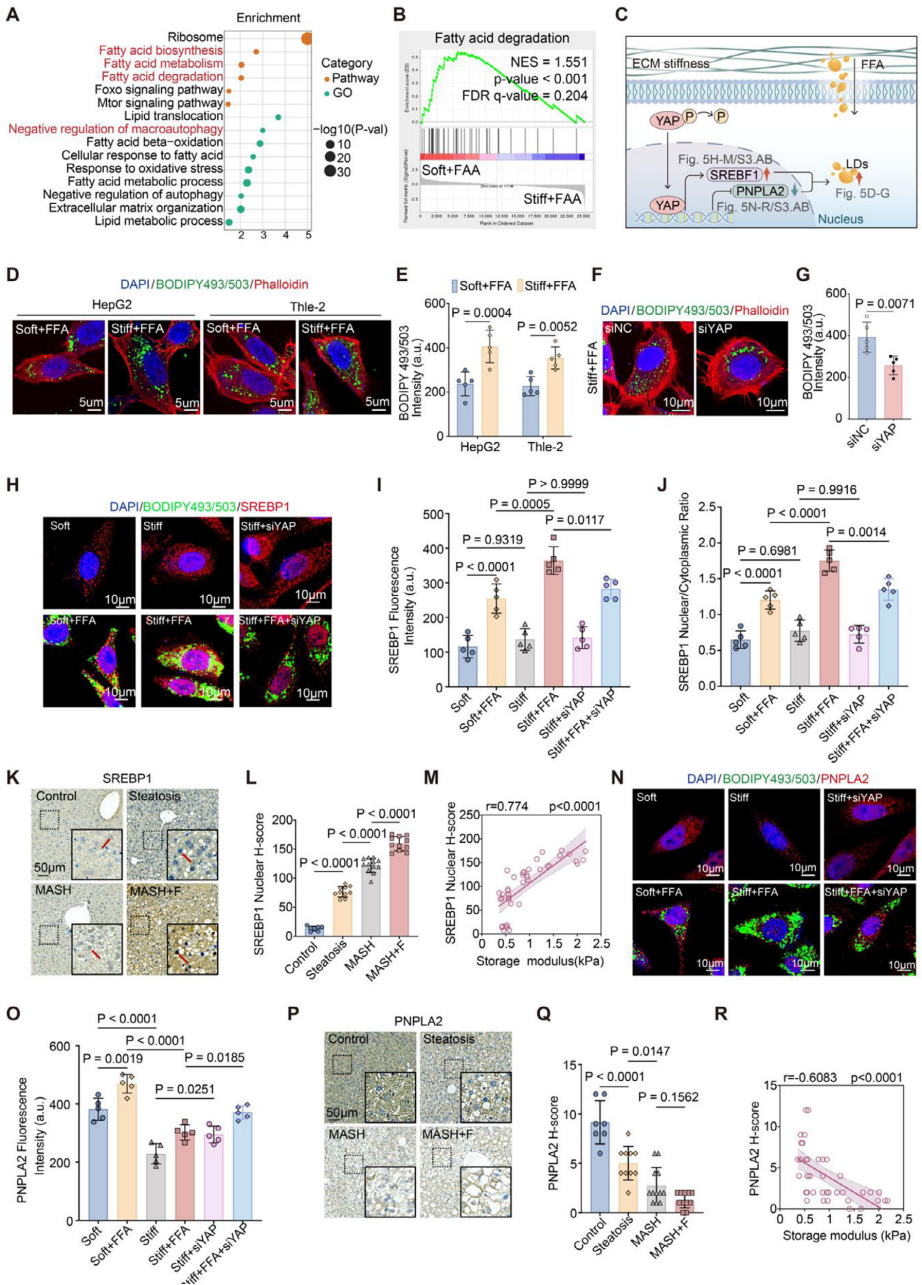
YAP links matrix stiffness to lipogenic reprogramming and suppression of lipolysis. (A) GO pathway enrichment analysis comparing stiff + FFA versus soft + FFA conditions in HepG2 cells. Stiff ECM combined with FFA loading significantly enriched pathways related to fatty acid biosynthesis, fatty acid metabolism, and fatty acid degradation, while also highlighting enrichment of negative regulation of macroautophagy, indicating coordinated activation of lipogenic programs and suppression of autophagic processes. (B) GSEA enrichment plot for the fatty acid degradation pathway based on the transcriptomic ranking of stiff + FFA versus soft + FFA conditions (NES = −1.551, *p* <0.001, FDR q = 0.204), showing a relative downregulation of fatty acid degradation under stiff mechanical cues combined with FFA exposure. (C) Schematic model illustrating how ECM stiffness and FFA cooperate through YAP to regulate LDs, upregulating lipogenesis (e.g., SREBP1) while repressing lipolysis (e.g., PNPLA2). (D,E) Representative immunofluorescence (IF) images of HepG2 and THLE‐2 cells showing LDs (BODIPY 493/503, green), F‐actin (phalloidin, red), and nuclei (DAPI, blue) under soft + FFA and stiff + FFA. Quantification of BODIPY fluorescence intensity shows significantly increased LD accumulation under stiff + FFA. Scale bars: 5 µm. (F,G) Representative IF images of HepG2 cells transfected with si‐NC or si‐YAP under stiff + FFA, showing reduced LD accumulation upon YAP knockdown. Scale bars: 10 µm. (H–J) IF staining of SREBP1 (red), BODIPY 493/503 (green), and DAPI (blue). YAP knockdown reduces SREBP1 nuclear accumulation under stiff conditions with or without FFA. Quantifications show fluorescence intensity (I) and N/C ratio (J). Scale bars: 10 µm. (K–M) IHC staining of SREBP1 in mouse liver tissues from control, steatosis, MASH, and fibrotic MASH (MASH+F). Quantification shows progressive increase of SREBP1 nuclear positivity (L) correlating with storage modulus (M, rheometry). Scale bars: 50 µm. (N,O) IF staining of PNPLA2 (red), BODIPY 493/503 (green), and DAPI (blue) in HepG2 cells. YAP knockdown restores PNPLA2 expression under stiff + FFA. Scale bars: 10 µm. (P–R) IHC staining of PNPLA2 in mouse liver tissues. PNPLA2 expression decreases with disease progression and negatively correlates with storage modulus. Scale bars: 50 µm. Data are mean ± SD (n = 3 independent biological replicates). Statistical significance was determined by one‐way ANOVA with Tukey's post hoc test or Spearman's correlation.

To assess how matrix stiffness modulates hepatocyte lipid‐related pathways under lipid overload, we performed pathway enrichment analysis focusing on lipid‐associated metabolic processes. Compared with the soft + FFA condition, the stiff + FFA condition showed significant enrichment of pathways involved in fatty acid biosynthesis, fatty acid metabolism, and fatty acid degradation (Figure 5A). GSEA further demonstrated a coordinated down‐enrichment of the fatty acid degradation pathway under stiff + FFA conditions (Figure 5B), indicating that under identical lipid‐loading conditions, increased matrix stiffness further suppresses transcriptional programs associated with fatty acid breakdown. Consistently, BODIPY staining demonstrated that stiff + FFA significantly enhanced LD accumulation in HepG2 and THLE‐2 cells compared with soft + FFA (Figure 5D,E). YAP knockdown markedly reduced LD deposition under stiff + FFA conditions (Figure 5F,G). Transcriptomic validation further confirmed that YAP silencing attenuated the induction of lipogenic genes (SREBP1, FASN, PLIN2) while restoring expression of the lipolytic mediator PNPLA2 (Figure S5A, B). Immunofluorescence analysis showed that stiff and stiff + FFA promoted SREBP1 nuclear translocation, which was blunted by YAP knockdown (Figure 5H–J; Figure S5C). In parallel, YAP silencing rescued PNPLA2 expression that was otherwise suppressed under stiff and stiff + FFA conditions (Figure 5N,O; Figure S5D). In vivo, IHC staining revealed progressive upregulation of nuclear SREBP1 with MASLD progression, positively correlating with hepatic storage modulus (Figure 5K–M), whereas PNPLA2 expression declined and negatively correlated with storage modulus (Figure 5P–R). These results demonstrate that ECM stiffening cooperates with LD overload through YAP to promote lipogenesis while suppressing lipolysis, thereby driving pathological LD accumulation (Figure 5C).

**FIGURE 6:**
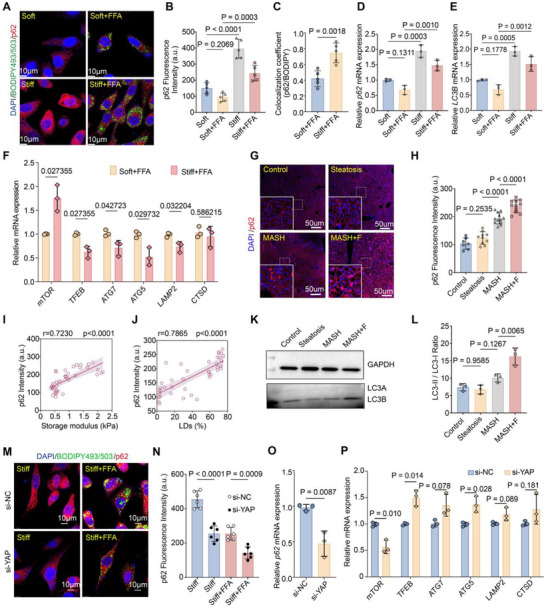
YAP mediates stiffness‐induced impairment of lipophagy. (A) Representative immunofluorescence (IF) images of HepG2 cells showing p62 (red), LDs (BODIPY 493/503, green), and nuclei (DAPI, blue) under Soft, soft + FFA, Stiff, and stiff + FFA conditions. Scale bars: 10 µm. (B) Quantification of p62 fluorescence intensity in HepG2 cells under the indicated conditions. (C) Colocalization coefficient of p62 puncta with BODIPY‐stained LDs under the indicated conditions. (D,E) qRT‐PCR analysis of *p62/SQSTM1* (D) and *LC3B* (E) mRNA expression under the indicated conditions. (F) qRT‐PCR analysis of core autophagy regulators (*mTOR, TFEB, ATG7, ATG5, LAMP2, CTSD*), showing no significant differences between soft + FFA and stiff + FFA. (G) Representative IF images of p62 (red) in mouse liver tissues from control, steatosis, MASH, and fibrotic MASH (MASH+F) groups. Nuclei are stained with DAPI (blue). Scale bars: 50 µm. (H) Quantification of p62 fluorescence intensity in mouse liver sections. (I,J) Correlations between hepatic p62 intensity and storage modulus (I), and between p62 intensity and LD burden (J). (K,L) Western blot analysis of LC3‐II and LC3‐I in mouse liver tissues, with quantification of the LC3‐II/LC3‐I ratio (L). GAPDH served as loading control. (M) Representative IF images of HepG2 cells showing p62 (red), BODIPY 493/503 (green), and nuclei (blue) under Stiff and stiff + FFA conditions with siNC or siYAP. Scale bars: 10 µm. (N–P) Quantification of p62 fluorescence intensity (N), *p62* mRNA expression (O), and selected autophagy regulators (*mTOR, TFEB, ATG7, ATG5, LAMP2, CTSD*) (P) in HepG2 cells with YAP knockdown. Data are mean ± SD (n = 3 independent biological replicates). Statistical significance was determined by one‐way ANOVA with post hoc tests for multiple comparisons or Spearman's correlation for associations.

Given that pathway analyses revealed suppression of fatty acid degradation and enrichment of negative regulation of macroautophagy under stiff + FFA conditions (Figure 5A,B). We next investigated whether autophagy, particularly lipophagy, was functionally impaired. In HepG2 cells, p62/SQSTM1 puncta accumulated on stiff substrates and further increased with FFA treatment (Figure 6A,B). Colocalization analysis revealed higher overlap of p62 with BODIPY‐labeled LDs under stiff + FFA (Figure 6C). qRT‐PCR showed upregulation of p62/SQSTM1 and LC3B, whereas core macroautophagy regulators (mTOR, TFEB, ATG7, ATG5, LAMP2, CTSD) remained unchanged (Figure 6D–F), suggesting a selective blockade of lipophagic flux rather than global autophagy. In MCD‐fed mouse livers, p62 expression increased progressively from simple steatosis to MASH and fibrotic MASH (Figure 6G,H), correlating positively with hepatic storage modulus and LD burden (Figure 6I,J). Western blotting confirmed elevated LC3‐II abundance and increased LC3‐II/I ratio in fibrotic MASH (Figure 6K,L). Functionally, *YAP* knockdown reduced p62 puncta and mRNA expression, and partially restored expression of selected autophagy regulators under both stiff and stiff + FFA conditions (Figure 6M–P). These findings indicate that YAP contributes to stiffness‐induced blockade of lipophagy, which cooperates with lipogenic reprogramming to promote LD accumulation.

Together, our data identify YAP as a central effector by which extracellular stiffness cooperates with FFA load to simultaneously enhance lipogenesis and suppress lipophagy. This dual effect drives pathological LD accumulation and reinforces mechano‐metabolic disease progression.

### 2.6 Piezo1 is An Upstream Mechanosensor Orchestrating Hepatocyte Responses to Stiffness

**FIGURE 7:**
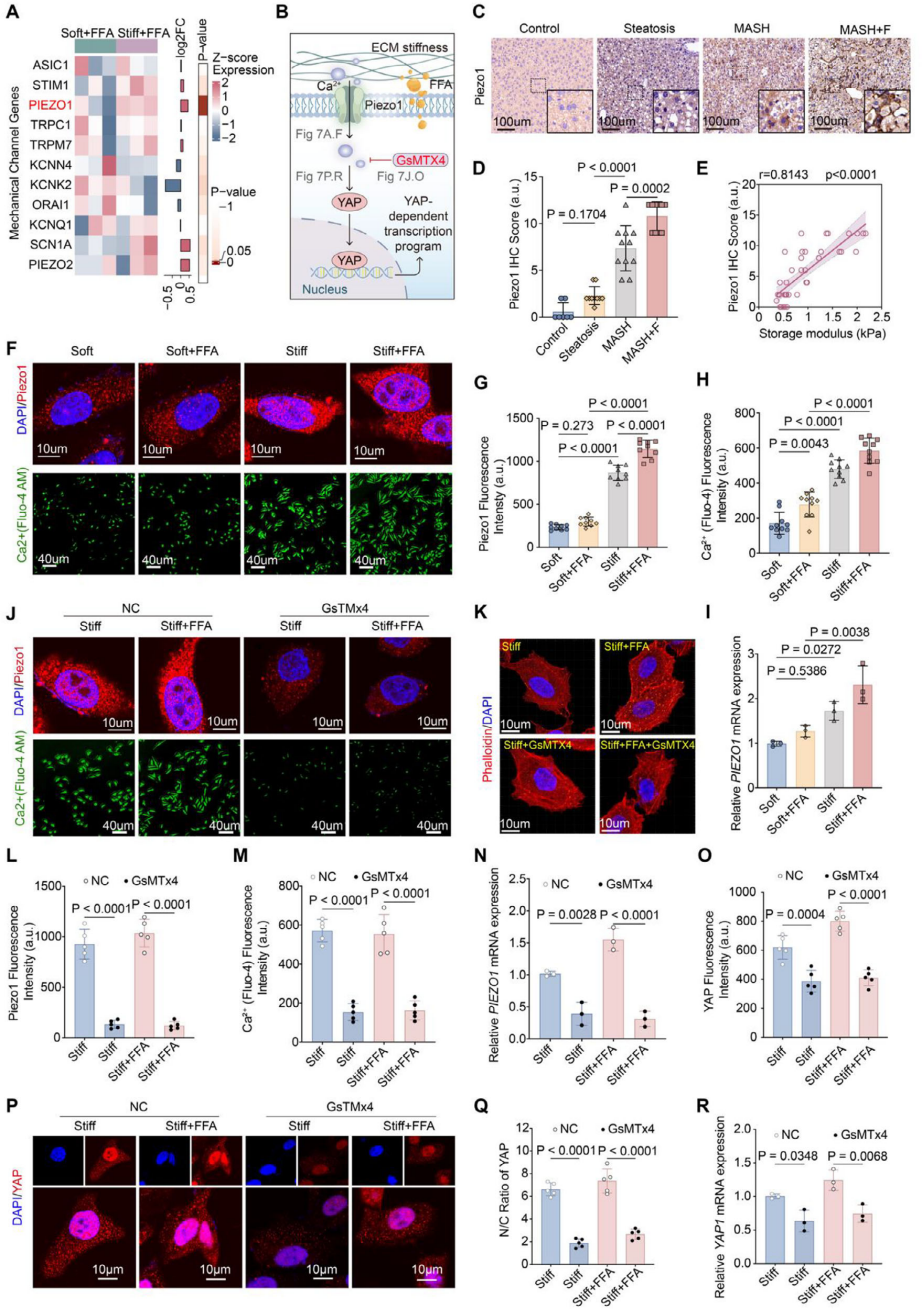
Piezo1 is an upstream mechanosensor orchestrating hepatocyte responses to stiffness. (A) Heatmap showing transcriptomic profiling of mechanosensitive ion channels under stiff + FFA versus soft + FFA conditions. Piezo1 was the most robustly induced, whereas other candidates (*ASIC1, TRPC1, KCNK4*) exhibited minimal changes. (B) Schematic diagram illustrating Piezo1 as an upstream mechanosensitive Ca^2^⁺ channel that links extracellular matrix stiffness to YAP‐dependent transcriptional programs. (C) Representative immunohistochemistry (IHC) staining of Piezo1 in mouse livers from control, steatosis, MASH, and fibrotic MASH (MASH+F). Scale bars: 100 µm. (D) Quantification of Piezo1 IHC score showing progressive upregulation from control and steatosis to MASH and MASH+F. (E) Correlation analysis between Piezo1 IHC score and hepatic storage modulus, showing a strong positive association (r = 0.8143, *p <*0.0001). (F) Representative immunofluorescence (IF) images of Piezo1 (red) and nuclei (DAPI, blue) in HepG2 cells cultured under Soft, soft + FFA, Stiff, and stiff + FFA conditions. Piezo1 exhibits a punctate or clustered distribution under stiff conditions. Scale bars: 10 µm. (G,H) Quantification of Piezo1 fluorescence intensity (G) and Ca^2^⁺ influx measured by Fluo‐4 AM staining (green, H) under the indicated conditions. Scale bars: 40 µm (Ca^2^⁺). (I) qRT‐PCR analysis of *PIEZO1* mRNA expression in HepG2 cells under the indicated conditions. (J–M) Pharmacological inhibition of Piezo1 with GsMTx4 under Stiff and stiff + FFA conditions. IF images show Piezo1 (red, J), Ca^2^⁺ influx (green, J), and actin cytoskeleton remodeling (phalloidin, K). Quantification of Piezo1 fluorescence (L), Ca^2^⁺ influx (M), and *PIEZO1* mRNA expression (N). (O–R) Effect of Piezo1 inhibition on YAP activity. IF images of YAP (red) and nuclei (blue) under the indicated conditions (P). Quantification of YAP fluorescence intensity (O), nuclear/cytoplasmic ratio of YAP (Q), and *YAP1* mRNA expression (R). Scale bars: 10 µm. Data are mean ± SD (n = 3 independent biological replicates). Statistical significance was determined by one‐way ANOVA with Tukey's post hoc test or Spearman's correlation.

Finally, to identify the mechanosensor upstream of YAP, we mined transcriptomic datasets from MASLD models and profiled a panel of mechanosensitive ion channels. Piezo1 was the most strongly induced under stiff + FFA conditions, whereas other candidates (ASIC1, TRPC1, KCNK4) changed minimally (Figure 7A,B).

In MCD diet‐fed mouse livers, Piezo1 expression increased progressively from control and simple steatosis to MASH and reached its highest levels in MASH with fibrosis. Quantitative analysis revealed a strong positive correlation between Piezo1 expression and hepatic storage modulus (r = 0.81, *p <*0.0001; Figure 7C–E). In vitro, stiff + FFA conditions induced clustered distribution of Piezo1 and robust Ca^2^⁺ influx in hepatocytes (Figure 7F–I). Pharmacological inhibition of Piezo1 with GsMTx4 (a peptide that primarily blocks Piezo1 but is not fully selective and may affect other mechanosensitive channels including Piezo2 and TRP family members at higher doses [25, 26]) decreased Piezo1 protein levels, blocked stiffness‐induced Ca^2^⁺ entry and *PIEZO1* mRNA upregulation, remodeled the actin cytoskeleton, and substantially reduced YAP activity, as evidenced by decreases in total YAP levels, nuclear localization, nuclear/cytoplasmic ratio, and *YAP1* mRNA expression (Figure 7J–R). These findings are consistent with Piezo1 functioning as an upstream mechanosensitive Ca^2^⁺ channel that mediates stiffness‐induced YAP activation, thereby orchestrating inflammatory, fibrogenic, and metabolic programs in hepatocytes.

## 3 Discussion

This study demonstrates that hepatic mechanical remodeling is an early and functionally critical feature of MASLD. Across two clinical cohorts, liver stiffness exhibited a modest but consistent upward trend with steatosis severity and was significantly higher in patients with biochemical evidence of inflammation; tissue‐scale AFM and rheometry in surgical specimens and MCD‐fed mice recapitulated these trends. These cross‐species findings challenge the prevailing view that elevated LSM primarily reflects advanced fibrosis [27, 28, 29] and instead establish that altered tissue mechanics is associated with early disease progression. Individuals with cirrhosis (F4) were rigorously excluded using validated LSM thresholds (≥14 kPa). Although subclinical F3 fibrosis cannot be fully ruled out in the absence of biopsy, segmented analyses helped minimize this confounding; stiffness preceded overt histological fibrosis in mouse models, supporting a dominant contribution from parenchymal and inflammatory remodeling. While the MCD diet lacks key metabolic features of human MASLD, such as obesity and insulin resistance [30, 31, 32], it reliably reproduces the full spectrum of steatosis, inflammation, and fibrosis, making it ideally suited for dissecting mechanobiological mechanisms. Notably, the Hertz model used for AFM assumes linear elasticity and material homogeneity; thus, AFM‐derived Young's moduli should be interpreted as apparent comparative parameters rather than absolute material properties. Accordingly, all pathophysiological correlations in this study were based on rheometry‐derived storage modulus, which more robustly captures tissue‐scale mechanics. Our findings are consistent with emerging evidence linking ECM physical properties to hepatic niche remodeling [15, 19, 33] and further introduce a previously underappreciated intracellular mechanism: mechanical stress arising from LD accumulation in hepatocytes. A concise mapping of these clinical phenotypes to their corresponding mechanistic validation is summarized in Table S5.

At the cellular level, previous studies have shown that stiff substrates reorganize F‐actin from branched networks into parallel stress fibers and transmit traction forces to the nucleus via the LINC/perinuclear actin cap axis [34, 35], whereas perinuclear clustering of LDs can deform the nucleus through direct compression [24]. Building on this framework, our study demonstrates that when ECM stiffening and FFA‐induced LD accumulation coexist, these external and internal mechanical cues synergize in hepatocytes to create conditions that promote transcriptional reprogramming and increase mechanotransductive sensitivity. Notably, malignant HepG2 cells exhibit greater nuclear surface expansion and loss of sphericity under stiff + FFA conditions, whereas non‐malignant THLE‐2 cells show more limited nuclear changes. This divergence highlights distinct mechanical thresholds and adaptive capabilities of the nuclear lamina–chromatin scaffold in different cellular contexts, with matrix stiffness acting as the dominant driver and lipid overload serving as a potentiator. Mechanistically, previous studies have shown that the formation of parallel stress‐fiber bundles involves tension‐dependent debranching of Arp2/3 actin networks and enhanced formin‐mediated polymerization, processes that together increase filament order and facilitate force transmission to the nucleus [36, 37]. These findings explain why stiff matrices, particularly in the presence of FFA‐driven steatosis, most effectively couple cytoskeletal remodeling to nuclear deformation, suggesting that validation in primary hepatocytes will be an important next step.

Within this framework, YAP functions as a mechanosensitive amplifier that transduces mechanical inputs into transcriptional outputs. Stiffness alone is sufficient to drive YAP nuclear translocation and pro‐fibrotic gene induction, whereas LD overload on stiff matrices further potentiates YAP‐dependent inflammatory signaling. Beyond inflammation and fibrosis, YAP integrates mechanical cues with lipid metabolism: stiff matrices enhance lipogenesis (*SREBP1*, *PLIN2*) and suppress lipolysis (*PNPLA2*, *CPT1A*), while *YAP1* knockdown reverses these changes and reduces LD burden. In parallel, stiff + FFA conditions selectively impair lipophagy, an effect alleviated by YAP silencing. These findings offer a mechanistic framework for pathological LD accumulation that, in turn, sustains inflammation and matrix deposition, thereby contributing to progressive tissue stiffening. The use of palmitate containing FFA mixtures raises the possibility of confounding lipotoxic effects; future studies with alternative fatty acid compositions will be required to fully separate mechanical from biochemical contribution. Finally, unlike prior work that primarily examined YAP activation in hepatic stellate cells or at the whole‐tissue mechanotransduction level [38, 39, 40, 41], this study identifies Piezo1 as the upstream mechanosensor in hepatocytes: it was strongly induced in MASH and MASH with fibrosis, correlated tightly with hepatic storage modulus, and mediated stiffness‐induced Ca^2^⁺ influx and YAP activation. Inhibition of Piezo1 with GsMTx4 abrogated Ca^2^⁺ entry, cytoskeletal remodeling, and YAP signaling, placing Piezo1–YAP at the core of hepatocyte mechano‐metabolic responses. Because GsMTx4 is not entirely specific for Piezo1 [42, 43], future studies employing genetic knockout models or next‐generation selective inhibitors will be needed to definitively confirm Piezo1 specificity. These findings support the proposed self‐reinforcing feed‐forward loop in early MASLD (Graphical Abstract). By incorporating mechanical inputs, lipid‐derived intracellular stress, and YAP‐dependent transcriptional outputs, our results extend current MASLD frameworks, traditionally centered on metabolic dysregulation and inflammation, into an integrated mechanical–metabolic–inflammatory axis [44, 45, 46, 47, 48]. Although cross‐scale evidence suggests that this vicious cycle might exist, its temporal dynamics and in vivo operation require confirmation in future longitudinal studies. Moreover, the molecular intermediates linking Piezo1‐mediated Ca^2^⁺ influx to actin cytoskeletal remodeling, nuclear envelope deformation, and subsequent YAP nuclear translocation remain poorly defined. Prior studies indicate that Ca^2^⁺‐sensitive regulators of actin dynamics, cytoskeleton–nucleus coupling through the LINC complex, and nuclear lamina mechanics may contribute to this process [49, 50, 51, 52], but their specific roles in hepatocytes remain elusive. Elucidating these downstream mechanotransduction steps represents an important and promising direction for future research.

Translationally, our findings indicate that LSM, beyond its conventional role in staging fibrosis, may serve as an early functional marker associated with metabolic stress and inflammation in MASLD. Patients with metabolic risk factors or active inflammation might be identified as having a window of early disease where mechanical alterations are already detectable. Second, a mechanism‐based therapeutic strategy emerges: (i) attenuate mechanical inputs (e.g., reduce collagen cross‐linking to soften the ECM or inhibit mechanosensitive channels like Piezo1), (ii) block downstream nuclear effectors (e.g., YAP/TEAD inhibitors), and (iii) restore healthy lipid handling (promote lipolysis and lipophagy). Although the Piezo1–YAP axis is firmly established in this study as a central mechanotransductive pathway, its therapeutic potential in MASLD patients spanning the full human metabolic spectrum, including obesity, insulin resistance, and dyslipidemia, will require further validation in complementary preclinical models that more faithfully recapitulate these metabolic features. LSM, alone or combined with emerging YAP‐related biomarkers, could serve as a practical, non‐invasive tool for patient selection and pharmacodynamic monitoring in early‐intervention MASLD trials.

## 4 Conclusion

This study reveals aberrant tissue mechanics as an early and active contributor to MASLD progression and delineates a novel mechanobiological axis linking ECM stiffness, lipid‐droplet‐derived stress, and Piezo1–YAP signaling in hepatocytes. We provide evidence that mechanical alterations associated with steatosis emerge early and activate specific mechanosensitive pathways that may exacerbate hepatocellular injury and promote fibrogenesis. Thus, abnormal tissue mechanics are not merely end‐stage hallmarks but active regulators that span inflammation, fibrosis, metabolic dysregulation, and impaired lipid‐droplet autophagy. A central advance of this work is the recognition of LDs as potent intracellular mechanical stressors. In parallel, our data position the Piezo1–YAP pathway as a key mechanotransductive node in hepatocytes, supporting an expanded MASLD framework that incorporates aberrant mechanotransduction alongside traditional metabolic and inflammatory drivers. Looking ahead, in vivo studies will be essential for validating this mechanobiological axis and for testing targeted interventions, such as Piezo1 inhibitors or matrix‐softening approaches, in relevant disease models. Extending this framework to other hepatic cell types (e.g., stellate cells, endothelial cells) and incorporating patient‐specific mechanical profiles will further refine our understanding of disease heterogeneity. Collectively, our findings support a mechanical–metabolic–inflammatory axis in early MASLD, in which physical forces from matrix stiffening and intracellular LDs may converge via Piezo1–YAP signaling to promote disease progression prior to irreversible fibrosis.

## 5 Materials and Methods

### 5.1 Clinical Sample Collection and Data Acquisition

Liver tissues were collected from surgical patients at the Second Affiliated Hospital of Xi'an Jiaotong University with informed consent. Demographic, laboratory, and imaging data were obtained from the Department of Gastroenterology. Liver stiffness (LSM) and controlled attenuation parameter (CAP) were measured using FibroScan, and AST or ALT >40 U/L was used as a surrogate indicator of hepatic inflammation. The study was approved by the hospital's Ethics Committee (No. 2022037). Baseline demographic and clinical characteristics of the cohorts are summarized in Tables S1 and S2.

Inclusion criteria:

1. Age between 18 and 75 years.
2. Clinical or imaging evidence of MASLD.
3. Completed blood biochemistry and Fibroscan examinations.

Exclusion criteria:

1. Excessive alcohol consumption (>210 g/week for men, >140 g/week for women).
2. Viral hepatitis (HBV/HCV), autoimmune liver disease, or drug‐induced liver injury.
3. Severe comorbidities (e.g., heart, kidney failure, malignancy).
4. Incomplete clinical data.
5. Definite cirrhosis (clinically overt decompensation, platelet count <150 × 10⁹/L, serum albumin <35 g/L, or LSM ≥14 kPa).

Note: Due to the absence of protocol liver biopsies, advanced fibrosis (F ≥3) could not be fully excluded.

### 5.2 Animal Model Establishment and Tissue Collection

Male C57BL/6J mice (5 weeks old, SPF grade; Laboratory Animal Center of Xi'an Jiaotong University) were acclimated for one week and then randomly assigned to either a control group fed standard chow, or an experimental group fed an MCD diet. Mice in the MCD group were euthanized at 2, 4, 6, or 8 weeks. Serum and liver tissues were collected for biomechanical testing, paraffin embedding, cryosectioning, and molecular analyses. Animal experiments were reviewed and approved by the IACUC of Xi'an Jiaotong University and performed following institutional regulations (XJTUAE2023‐1434).

### 5.3 Atomic Force Microscopy (AFM) Measurements

Liver tissues were placed in optimal cutting temperature (OCT) compound, overnight freezing at −80 °C, and cryostat sectioning (100‐µm thickness, Leica CM1950). To provide structural support, the tissue surface was covered with a polymer film (Cryofilm type II C, Section‐Lab, Japan; or Cryojane Tape Window, Leica, Germany) prior to sectioning. After cutting, the cryofilm with adherent tissue was transferred to a 35‐mm dish, crosslinked under UV light for 30 s, and trimmed of excess film. Sections were maintained in phosphate‐buffered saline (PBS) during AFM analysis to prevent degradation.

Indentation experiments were conducted with a Nanowizard IV Bio‐AFM (JPK Instruments, Germany) integrated into an inverted fluorescence microscope (Nikon Eclipse Ti2) for real‐time positioning, with a temperature‐controlled stage maintained at 37 °C. For both liver tissue sections and adherent hepatocytes, NPO‐O10 cantilevers (type B, spring constant ∼0.12 N/m) and cantilevers modified with a 12 µm polystyrene bead were used in contact mode force measurements.

For all samples (tissue and cells), indentation was performed at a constant loading rate of 2 µm/s (z‐ramp size 8 µm, maximum trigger force 5 nN), corresponding to an effective strain rate of approximately 0.2–0.6 s^−1^ depending on indentation depth. This rate is widely used for soft biological materials to limit pronounced viscoelastic effects while maintaining reproducible force–distance curves. Liver tissue: maximum indentation depth was limited to ≤800 nm to avoid substrate effects and preserve linear Hertzian contact. Cultured hepatocytes: indentation depth was restricted to ≤500–700 nm (≤10–15% of cell height) to ensure measurements remained within the small‐strain regime and avoided nuclear indentation artifacts.

Force–distance curves were processed with JPK software and fitted to the Hertz model (Poisson's ratio = 0.5) to calculate the apparent Young's modulus. At least 12 force curves were recorded per region, with ≥8 regions per sample and n ≥5 independent biological replicates per group. Because this model assumes linear elasticity and material homogeneity, all extracted apparent Young's moduli are reported as apparent values, suitable for comparative analysis rather than absolute mechanical properties, consistent with established practices for soft, hydrated, and viscoelastic biological tissues.

### 5.4 Rheometry

Bulk viscoelastic properties of liver tissue were assessed by oscillatory shear rheometry. Experiments were conducted at 37.0  ±  0.1 °C on a stress‐controlled Discovery HR‐2 or DHR‐3 rheometer (TA Instruments) using 8 mm diameter parallel‐plate geometry with serrated surfaces and a gap height of 1000 µm to ensure consistent sample contact. Liver tissue samples were prepared as ∼1 mm‐thick discs (∼4 mm diameter) with parallel smooth surfaces. The liver capsule, connective tissue, and large vessels were removed to minimize heterogeneity artifacts.

To ensure that measurements were performed within the linear viscoelastic regime (LVR), strain amplitude sweeps (0.1–10% strain at 1 Hz) were conducted on representative samples from each experimental group. These sweeps confirmed that both G′ and G″ remained strain‐independent up to approximately 5–10% strain. Based on these results, an oscillatory strain of 1% was selected as a conservative value well within the LVR.

An initial normal force of 0.03–0.08 N was applied, and samples were equilibrated at 37 °C for 1 min before testing. Storage modulus was measured under oscillatory shear (0.1–10 Hz, 1% strain) in triplicate and averaged.

### 5.5 Cell Culture and Biomechanical Microenvironment Simulation

Polyacrylamide (PA) substrates of defined stiffness were prepared by adjusting the acrylamide and bis‐acrylamide composition. Briefly, stock solutions of 40% (w/v) acrylamide and 2% (w/v) bis‐acrylamide were mixed at defined ratios (final volume 100–200 µL) and polymerized with 10% ammonium persulfate (2 µL) and TEMED (0.2 µL) for 30 min on glass coverslips (1 or 3 cm diameter), yielding PA gel substrates ∼100 µm thick. To facilitate cellular attachment, sulfo‐SANPAH (1 mg/mL in HEPES buffer) was applied onto the gel surface and activated under UV light, then washed with HEPES buffer to remove excess reagent. Type I collagen (0.1 mg/mL) was conjugated to the activated gel surface. Hydrogels were stored in PBS and sterilized by UV for 30 min prior to cell seeding.

### 5.6 Cell Culture and Treatment

HepG2 and THLE‐2 cells (ATCC) were grown in high‐glucose DMEM containing 10% fetal bovine serum and 1% penicillin–streptomycin at 37 °C in 5% CO_2_. For stiffness and lipid‐loading assays, approximately 1 × 10^5 cells per well were seeded onto collagen‐coated polyacrylamide hydrogels and allowed to adhere for 24 h. Intracellular LD accumulation was then induced by treating the cells for 24 h with a 2:1 mixture of oleic acid and palmitic acid at a final FFA concentration of 0.5 mm.

### 5.7 Immunofluorescence Staining

Cells were fixed, permeabilized, blocked, and incubated with primary and fluorophore‐conjugated secondary antibodies; nuclei were counterstained with DAPI. Frozen liver tissues were OCT‐embedded, cryosectioned (8–10 µm), and processed similarly. Images were acquired with an Olympus FV3000 confocal microscope. Fluorescence intensity was quantified in 30–50 cells per group from three independent experiments using ImageJ, with uniform thresholds applied for positive cell determination. Representative 60× images were shown, with imaging parameters kept constant across groups. The antibodies used are shown in Table S3. All staining experiments were performed with 3 biological replicates and analyzed with the software Image J.

### 5.8 Quantitative Real‐Time PCR (qRT‐PCR)

Total RNA was isolated from cultured cells using TRIzol reagent (Invitrogen, USA). cDNA was synthesized with the PrimeScript RT kit (YEASEN, China), and quantitative real‐time PCR was performed using SYBR Green Master Mix (YEASEN, China) on a StepOnePlus Real‐Time PCR System (Applied Biosystems, USA). GAPDH served as the internal control, and relative expression was calculated using the 2^−ΔΔCt^ method. The qPCR primers used are shown in Table S4.

### 5.9 Statistical Analysis

“n” represents independent biological replicates. Data are presented as mean ± SD. Differences among multiple groups were analyzed using one‐way ANOVA followed by Tukey's post hoc test when data were normally distributed, or the Kruskal–Wallis test followed by Dunn's post hoc test when normality was not met. Correlations were assessed using Spearman's rank correlation coefficient. Two‐tailed *p* values < 0.05 were considered statistically significant. GraphPad Prism 9.0 (GraphPad Software, USA) was used for cell/mouse data, and SPSS 28.0 (IBM, USA) was used for clinical datasets.

## Author Contributions

N.L., H.S., and F.X. conceived and designed the study. J.M. was responsible for data curation, investigation, and formal analysis. N.X., Z.W., and X.L. performed visualization, including figure preparation and editing. H.L. and W.K. provided resources, specifically the collection of liver tissues. M.W. developed and performed the methodology for AFM measurements. Ning L. contributed to language editing. All authors discussed the results and wrote the paper.

## Conflicts of Interest

The authors declare no conflicts of interest.

## Supporting information




**Supporting File**: advs73533‐sup‐0001‐SuppMat.docx.

## Data Availability

The data that support the findings of this study are available from the corresponding author upon reasonable request.
