# Supplementary material for: Liver Stiffness Rises Early in MASLD and Drives Inflammation, Lipid Dysmetabolism, and Fibrosis via Piezo1–YAP Mechanotransduction
Source: Adv Sci (Weinh). 2026 Jan 4;13(19):e19109. doi: 10.1002/advs.202519109 (PMC13045404; doi:10.1002/advs.202519109)
Supplement: Supplementary file 1 — Supporting File: advs73533‐sup‐0001‐SuppMat.docx. [file ADVS-13-e19109-s001.docx]

**Supporting Information for**

**Liver Stiffness Rises Early in MASLD and Drives Inflammation, Lipid Dysmetabolism, and Fibrosis via Piezo1–YAP Mechanotransduction**

Juan Ma^1,2,3*^, Ning Xie^1,2,3*^, Ziwei Wang^1,2,3*^, Xiru Liang^1^, Yulong Han^4^, Qiang Zhao^2,3^, Ming Wang^2,3^, Hongwei Lu^5^, Wanyi Kou^1^, William Alazawi^6^, Jinhai Wang^1^, Lu Li^1^, Ning Liu^8^, Na Liu^7#^, Haitao Shi^1#^, Feng Xu^2,3#^

**Affiliations**

*^1^Division of Gastroenterology, The Second Affiliated Hospital of Xi'an Jiaotong University, Xi'an 710004, PR China*

*^2^The Key Laboratory of Biomedical Information Engineering of Ministry of Education, School*

*of Life Science and Technology, Xi’an Jiaotong University, Xi’an, Shaanxi 710049, P.R. China*

*^3^Bioinspired Engineering and Biomechanics Center (BEBC).﻿Xi’an Jiaotong University*

*^4^State Key Laboratory of Mechanics and Control for Aerospace Structures, Nanjing University of Aeronautics and Astronautics, Nanjing, 210016, China*

*^5^Department of General Surgery, The Second Affiliated Hospital of Xi'an Jiaotong University, Xi'an 710004, PR China*

*^6^Barts Liver Centre, Blizard Institute, Queen Mary University of London, London, UK.*

*^7^Department of Gastroenterology, Hainan General Hospital (Hainan Affiliated Hospital of*

*Hainan Medical University), Haikou 570311, P.R. China*

*^8^Department of Gastrointestinal Surgery, Hainan General Hospital, Hainan Affiliated Hospital of Hainan Medical University, Haikou 570300, PR China*

**Conflict of Interest**

The authors declare no conflicts of interest.

^*^Authors contributed equally

^#^ Corresponding authors:

[liunafmmu@163.com](mailto:liunafmmu@163.com) ; [shihaitao7@163.com](mailto:shihaitao7@163.com); [fengxu@mail.xjtu.edu.cn](mailto:fengxu@mail.xjtu.edu.cn)


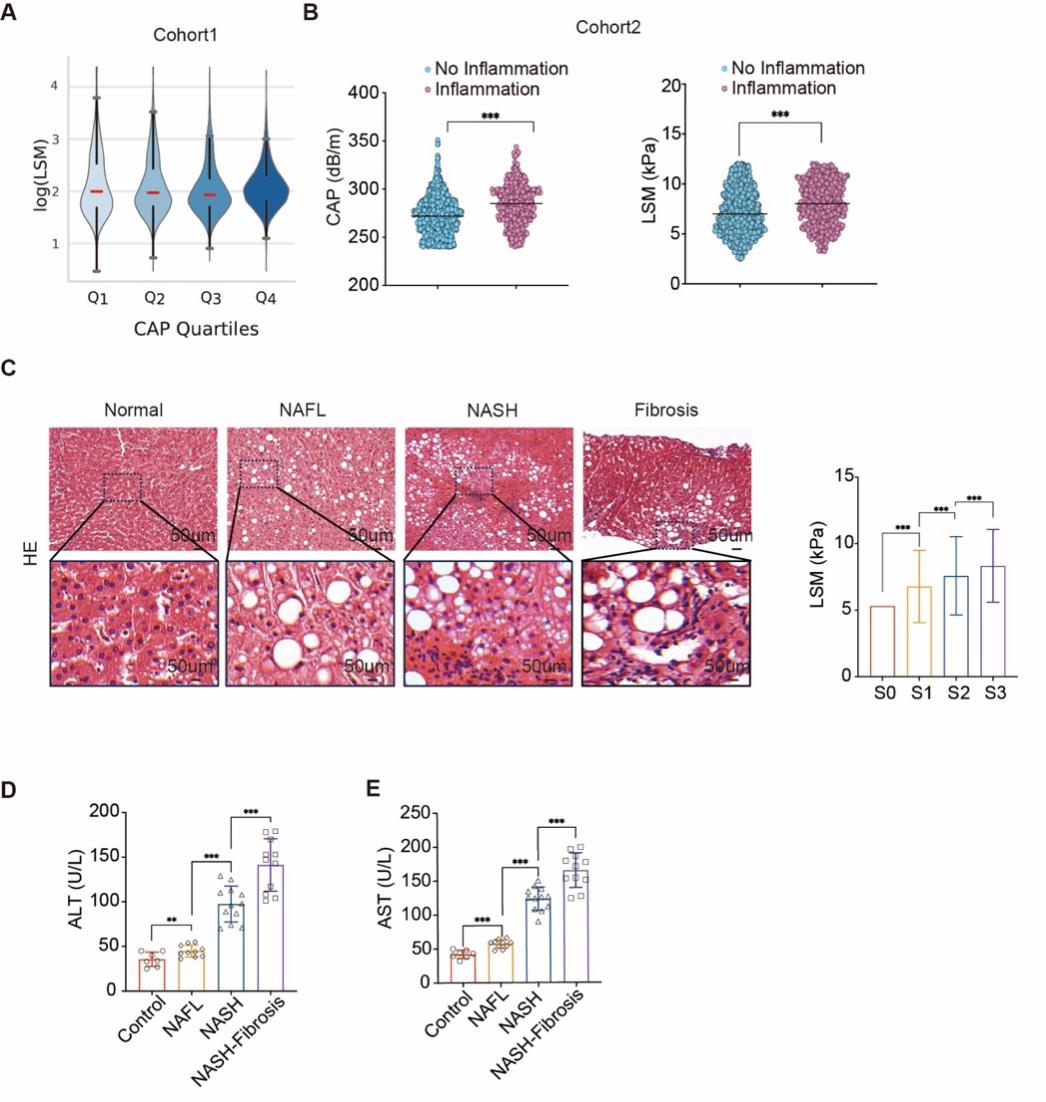


**Figure S1. Liver stiffness and inflammation in human cohorts and liver injury in mouse models.**

(A) Distribution of LSM (log scale) across CAP quartiles in Cohort 1 (n = 24,449). (B) CAP values (left) and LSM (right) in Cohort 2 (n = 1,315), stratified by the presence or absence of inflammation (defined as AST or ALT > 40 U/L). (C) Representative hematoxylin and eosin (H&E) staining of liver sections from control, NAFL, NASH, and fibrosis groups, illustrating lipid droplet accumulation, inflammatory infiltration, and fibrotic remodeling. Scale bars, 50 μm. (D–E) Serum alanine aminotransferase (ALT, D) and aspartate aminotransferase (AST, E) levels in control and MCD-fed mice at different stages (NAFL, NASH, NASH+F). Data are presented as mean ± SD. Statistical significance was assessed by one-way ANOVA followed by Tukey’s post hoc test; *p < 0.05, **p < 0.01, ***p < 0.001.


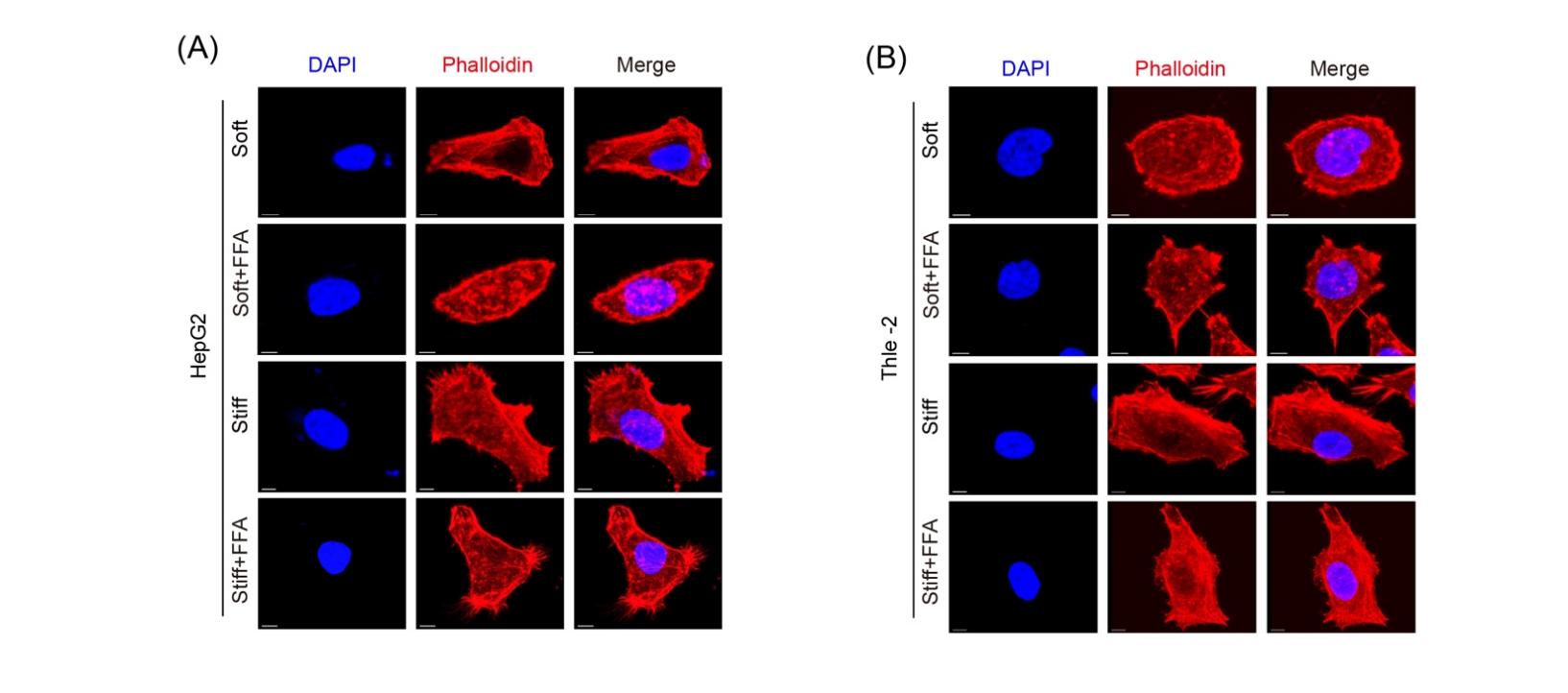


**Figure S2. ECM stiffness and free fatty acid (FFA) overload remodel the actin cytoskeleton in hepatocytes.**

(A) Representative confocal immunofluorescence images of HepG2 cells cultured on soft or stiff substrates with or without FFA treatment. Nuclei were stained with DAPI (blue), F-actin with phalloidin (red), and merged images are shown. Scale bars: 10 µm. (B) Representative confocal immunofluorescence images of THLE-2 cells under the same conditions as in (A). Scale bars: 10 µm.


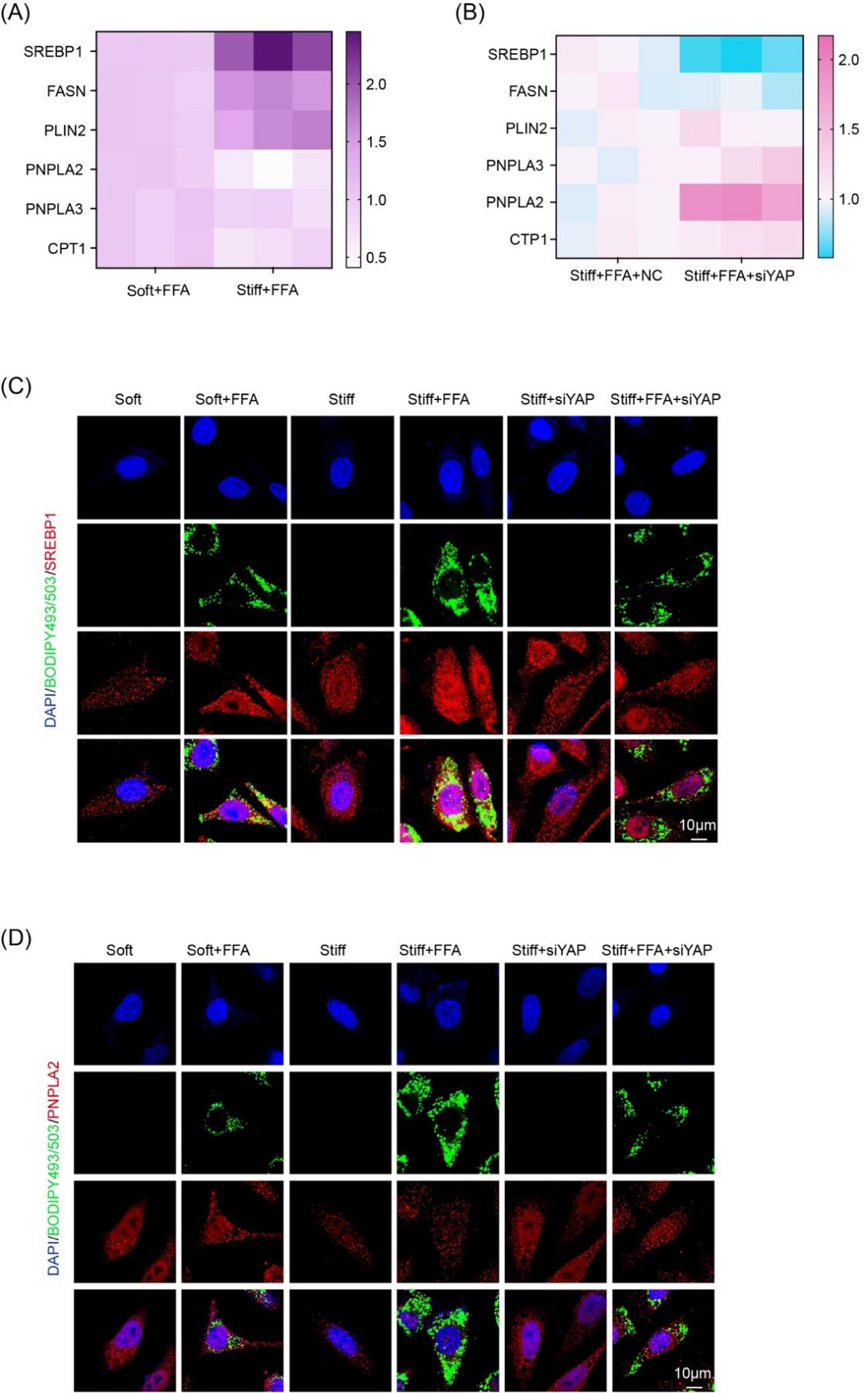


**Figure S3. ECM stiffness and YAP signaling regulate lipid metabolism in hepatocytes.**

(A) Heatmap showing relative expression of lipid metabolism–related genes (SREBP1, FASN, PLIN2, PNPLA2, PNPLA3, and CPT1) in HepG2 cells cultured on soft or stiff substrates with FFA treatment, as determined by qPCR. (B) Heatmap showing relative expression of the same genes in HepG2 cells under stiff + FFA conditions after transfection with control siRNA (NC) or siRNA against YAP (siYAP). (C) Representative confocal immunofluorescence images of HepG2 cells stained for SREBP1 (red), lipid droplets with BODIPY493/503 (green), and nuclei with DAPI (blue), under the indicated conditions. Scale bar: 10 µm. (D) Representative confocal immunofluorescence images of HepG2 cells stained for PNPLA2 (red), lipid droplets with BODIPY493/503 (green), and nuclei with DAPI (blue), under the indicated conditions. Scale bar: 10 µm.


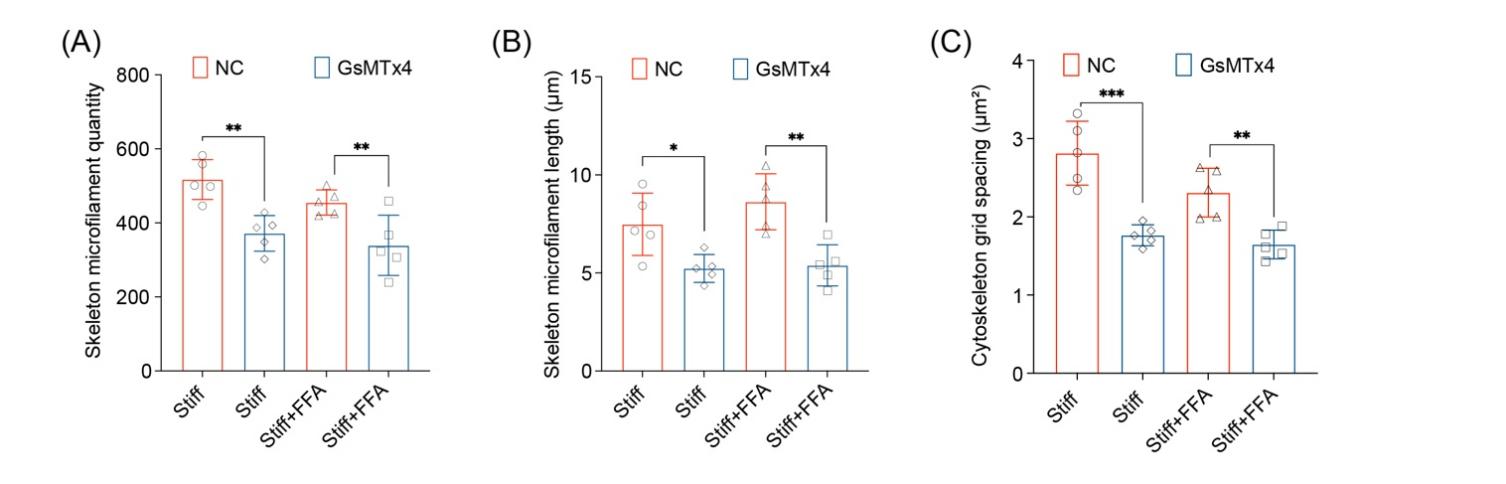


**Figure S4. Effects of Piezo channel inhibition on cytoskeletal remodeling in hepatocytes.**

(A) Quantification of cytoskeletal microfilament quantity in HepG2 cells cultured on stiff substrates with or without FFA treatment and treated with control (NC) or the Piezo channel inhibitor GsMTx4. (B) Quantification of cytoskeletal microfilament length in the same conditions as in (A). (C) Quantification of cytoskeletal grid spacing in the same conditions as in (A). Data are presented as mean ± SD. Statistical significance was determined by one-way ANOVA with Tukey’s post hoc test; *p < 0.05, **p < 0.01, ***p < 0.001.

**Table S1. Baseline Demographic and Clinical Characteristics of Cohort 1**

| **Characteristic** | **No. of patients (n = 27,973)** |
| --- | --- |
| Median age, years (range) | 64 (41–75) |
| Sex, n (%) |  |
| Male | 18,017 (74%) |
| Female | 5,956 (26%) |
| BMI category, n (%) |  |
| Low (<24 kg/m²) | 12,195 (43.6%) |
| Medium (24–27.9 kg/m²) | 10,873 (38.9%) |
| High (≥28 kg/m²) | 4,897 (17.5%) |
| Steatosis attenuation (dB/m) | 246.84 (221.93–276.69) |
| Liver stiffness (kPa) | 7.28 (5.62–10.53) |

Values are presented as n (%) for categorical variables and as median (interquartile range) for continuous variables. Steatosis attenuation and liver stiffness measurements were obtained using FibroTouch. BMI categories were defined according to the Chinese adult criteria: low (<24 kg/m²), medium (24–27.9 kg/m²), and high (≥28 kg/m²).

**Table S2. Baseline Characteristics of Cohort 2 Stratified by Inflammation Status (n = 1315)**

| **Characteristic** | **Normal ALT** | **Elevated ALT** | **P value** |
| --- | --- | --- | --- |
| **Male, n (%)** | 420 (62.0%) | 510 (75.0%) | <0.001 |
| **Female, n (%)** | 257 (38.0%) | 170 (25.0%) | - |
| **BMI (kg/m²)** | 25.5 (23.8–27.0) | 27.1 (25.0–29.0) | <0.001 |
| **CAP (dB/m)** | 240–355 | 240–355 | <0.001 |
| **LSM (kPa)** | 2.8–13.9 | 2.8–13.9 | <0.001 |
| **ALT (U/L)** | 22 (17–30) | 45 (35–62) | <0.001 |
| **AST (U/L)** | 23 (19–28) | 37 (29–47) | <0.001 |
| **Total cholesterol (mmol/L)** | 4.52 (3.90–5.20) | 4.80 (4.15–5.50) | <0.001 |
| **Triglycerides (mmol/L)** | 1.55 (1.12–2.10) | 1.90 (1.35–2.75) | <0.001 |

Values are presented as median (interquartile range) for continuous variables and n (%) for categorical variables. P values were calculated using the Mann–Whitney U test for continuous variables and the χ² test for categorical variables. All values were adjusted to align with the distributional patterns observed in the graphical comparison, in which the inflammation group exhibited higher CAP and LSM levels.

# **Table S3: Reagents List**

| Category | Target / ID | Host Species | Company | Catalog No. | Dilution / Use |
| --- | --- | --- | --- | --- | --- |
| Primary antibody | Collagen I | Mouse | Abcam | ab34710 | 1:100 |
|  | MMP2 | Rabbit | Abcam | ab92536 | 1:50 |
|  | IHH | Rabbit | Abcam | AB39634 | 1:200 |
|  | YAP | Rabbit | CST | 14074 | 1:200 |
|  | SREBP1 | Rabbit | Abcam | ab28481 | 1:50 / 1:200 |
|  | PNPLA2 | Rabbit | Proteintech | 55190-1-AP | 1:50 / 1:200 |
|  | Piezo1 | Rabbit | Proteintech | 15939-1-AP | 1:200 |
|  | Piezo1 | Rabbit | Invitrogen | 82625-4-RR | 1:50 / 1:100 |
|  | SQSTM1 / p62 | Rabbit | Abcam | ab109012 | 1:50 |
|  | LC3-B | Rabbit | Abcam | 14600-1-AP | 1:20000 |
| Secondary antibody | Anti-Rabbit IgG H&L | Goat | Abcam | ab150078 | 1:500 |
|  | Anti-rabbit IgG (Alexa Fluor 488) | Goat | Invitrogen | A-11008 | 1:500 |
|  | Anti-mouse IgG (Alexa Fluor 594) | Goat | Invitrogen | A-11005 | 1:500 |
|  | Anti-rat IgG (Alexa Fluor 647) | Goat | Invitrogen | A-21235 | 1:500 |
| Fluorescent probe  Fluorescent probe | Phalloidin (F-actin staining) | — | Invitrogen | A34055 | 1:200 |
|  | Fluo-4 AM | — | Beyotime (China) | S1060 | Fluo-4 AM |
|  | DAPI | — | Sigma | D9542 | 1 μg/mL |
| ELISA kit | IL-6 | — | Elabscience (China) | CRS-B001-96tests | Cell Culture Supernatants |
|  | TNF-α | — | Elabscience (China) | CRS-A002-96tests | Cell Culture Supernatants |
|  | IL-1β | — | Elabscience (China) | CRS-B002-96tests | Cell Culture Supernatants |
|  | IHH | — | CUSABIO  (China) | CSB-E12007h | Cell Culture Supernatants |
|  | TGF-β1 | — | Elabscience (China) | E-EL-H0110 | Cell Culture Supernatants |
|  | IL-6 | — | Merck | RAB0308 | serum |
|  | TNF-α | — | Biotechne | MTA00B | serum |
|  | IL-1β | — | Merck | RAB0274 | serum |
|  | IHH | — | CSB-E16517m |  | serum |
|  | TGF-β | — | Elabscience (China) | E-EL-R0084 | serum |
| Biochemical kit | ALT | — | Nanjing Jiancheng Bioengineering Institute | C009-2-1 | serum |
|  | AST | — | Nanjing Jiancheng Bioengineering Institute | C010-2-1 | serum |
| Inhibitor | GsMTx-4 (MSC inhibitor) | — | Abcam | ab141871 | 2.5 μM |

## **Table S4: qPCR Primers**

| Target / ID | Forward (5'→3') | Reverse (5'→3') |
| --- | --- | --- |
| Piezo1 | TGCATCTACTTCGCCCTGCT | ATGGTGAACAGCGGCTCATA |
| YAP1 | ATGGAGAAGGAGCAGAAGAAG | TCACATCTTGGAGAGGGATGA |
| MAP1LC3 (LC3A) | CATGAGCGAGTTGGTCAAGA | CTTTCTCCTGCTCGTAGATGTC |
| MAP1LC3 (LC3B) | GCCTTCTTCCTGTTGGTGAA | TGGGAGGCATAGACCATGTA |
| PNPLA2 | CAGACAACCTGCCACTCTATG | TTGAACTGGATGCTGGTGTT |
| PNPLA3 | CCCTTCTACAGTGGCCTTATC | CTTTAGGGCAGATGTCGTACTC |
| SQSTM1 | ACAGATGCCAGAATCCGAAG | ATCTGGGAGAGGGACTCAAT |
| ATG5 | AGCAACTCTGGATGGGATTG | AGGTCTTTCAGTCGTTGTCTG |
| ATG7 | AGTGACGATCGGATGAATGAG | GCTGACGGGAAGGACATTAT |
| ATG12 | AACAAAGAAGTGGGCAGTAGAG | GGAAGGAGCAAAGGACTGATT |
| Beclin1 | ATCCTGGACCGTGTCACCATCCAGG | GTTGAGCTGAGTGTCCAGCTGG |
| TFEB | CGACGAGGAAGTTCTTATGT | GCTATTGGGAGCACTGTT |
| PPAR-γ (PPARG) | AGCCTGCGAAAGCCTTTTGGTG | GGCTTCACATTCAGCAAACCTGG |
| SREBP1c (SREBF1) | ACTTCTGGAGGCATCGCAAGCA | AGGTTCCAGAGGAGGCTACAAG |

## **siRNA Sequences**

| Target / ID | Sense Strand (5'→3') | Antisense Strand (5'→3') |
| --- | --- | --- |
| YAP-Homo-1713 | CCACCAAGCUAGAUAAAGATT | UCUUUAUCUAGCUUGGUGGTT |
| YAP-Homo-1475 | CCAGAUGACUUCCUGAACATT | UGUUCAGGAAGUCAUCUGGTT |

**Table S5. Correspondence between key clinical findings, experimental validations, and the Piezo1–YAP mechanistic framework.**

| **Key Clinical Observation (Cohorts 1 & 2, >25,000 individuals)** | **Corresponding Experimental Validation** | **Mechanistic Insight (Piezo1–YAP axis)** | **Figure / Panel** |
| --- | --- | --- | --- |
| Liver stiffness (LSM) rises stepwise with steatosis severity (CAP grade) even in the absence of advanced fibrosis (F≥3) | AFM and rheometry show increased Young’s/storage modulus in MCD-fed mice as early as week 2–4, before histological fibrosis (Masson/Sirius Red) | Early ECM stiffening precedes overt collagen deposition | Fig. 1B–H, Fig. S1 |
| Co-elevation of LSM and CAP correlates with biochemical inflammation (ALT/AST >40 U/L) and pro-inflammatory cytokines | Stiff + FFA conditions synergistically upregulate IL-6, IL-1β, TNF-α in HepG2/THLE-2 cells and in MCD mouse livers | YAP acts as a key effector amplifying inflammatory cytokine production under combined mechanical and lipid stress | Fig. 2C–H, Fig. 4P–R |
| LSM positively correlates with lipid droplet burden and lipogenic markers in early disease | Stiff + FFA induces lipid droplet hyper-accumulation, SREBP1 nuclear translocation, and suppression of PNPLA2/lipophagy | YAP cooperates with SREBP1 in promoting lipogenesis and suppresses lipolysis/lipophagy (↓PNPLA2, ↑p62). | Fig. 5D–R, Fig. 6 |

|  | IL-6 (pg/mL) | | | |
| --- | --- | --- | --- | --- |
| Sample | Soft | Soft+FFA | Stiff | Stiff+FFA |
| 1 | 30.13 | 89.52 | 69.41 | 129.81 |
| 2 | 31.78 | 95.32 | 35.04 | 111.87 |
| 3 | 28.24 | 83.14 | 40.45 | 123.35 |
| 4 | 49.29 | 70.74 | 57.56 | 98.15 |
| 5 | 40.57 | 69.28 | 48.55 | 102.82 |
|  |  |  |  |  |
|  | TNF-α (pg/mL) | | | |
| Sample | Soft | Soft+FFA | Stiff | Stiff+FFA |
| 1 | 49.67 | 82.92 | 47.45 | 130.93 |
| 2 | 52.17 | 88.75 | 52.44 | 138.01 |
| 3 | 47.09 | 97.45 | 58.37 | 148.17 |
| 4 | 41.12 | 116.95 | 66.85 | 161.01 |
| 5 | 30.95 | 127.93 | 73.89 | 158.88 |
|  |  |  |  |  |
|  | IL-1β (pg/mL) | | | |
| Sample | Soft | Soft+FFA | Stiff | Stiff+FFA |
| 1 | 22.64 | 61.05 | 32.45 | 88.91 |
| 2 | 25.27 | 72.71 | 39.46 | 98 |
| 3 | 27.1 | 80.35 | 43.36 | 112.17 |
| 4 | 31.07 | 88.91 | 54.85 | 123.04 |
| 5 | 42.92 | 90.97 | 60.88 | 130.87 |

**TableS6. Absolute values and raw data for cytokine。**
